# Spatial and Functional Distribution of *MYBPC3* Pathogenic Variants and Clinical Outcomes in Patients With Hypertrophic Cardiomyopathy

**DOI:** 10.1161/CIRCGEN.120.002929

**Published:** 2020-08-25

**Authors:** Adam S. Helms, Andrea D. Thompson, Amelia A. Glazier, Neha Hafeez, Samat Kabani, Juliani Rodriguez, Jaime M. Yob, Helen Woolcock, Francesco Mazzarotto, Neal K. Lakdawala, Samuel G. Wittekind, Alexandre C. Pereira, Daniel L. Jacoby, Steven D. Colan, Euan A. Ashley, Sara Saberi, James S. Ware, Jodie Ingles, Christopher Semsarian, Michelle Michels, Iacopo Olivotto, Carolyn Y. Ho, Sharlene M. Day

**Affiliations:** 1 Cardiovascular Medicine (A.S.H., A.D.T., N.H., S.K., J.R., J.M.Y., H.W., S.S.), University of Michigan, Ann Arbor.; 2 Molecular & Integrative Physiology (A.A.G.), University of Michigan, Ann Arbor.; 3 Department of Experimental & Clinical Medicine, University of Florence, Italy (F.M., I.O.).; 4 National Heart & Lung Institute & Royal Brompton Cardiovascular Research Center, Imperial College London, United Kingdom (F.M., J.S.W.).; 5 Cardiovascular Medicine, Brigham & Women’s Hospital, Harvard Medical School, Boston, MA (N.K.L., C.Y.H.).; 6 Cincinnati Children’s Hospital Medical Center, Heart Institute, Cincinnati, OH (S.G.W.).; 7 Heart Institute (InCor), University of Sao Paolo Medical School, Brazil (A.C.P.).; 8 Cardiovascular Medicine, Yale University, New Haven, CT (D.L.J.).; 9 Department of Cardiology, Boston Children’s Hospital, MA (S.D.C.).; 10 Center for Inherited Heart Disease, Stanford University, CA (E.A.A.).; 11 Agnes Ginges Centre for Molecular Cardiology at Centenary Institute, The University of Sydney, Australia (J.I., C.S.).; 12 Department of Cardiology, Erasmus Medical Center, Rotterdam, the Netherlands (M.M.).; 13 Cardiomyopathy Unit, Careggi University Hospital, Florence, Italy (I.O.).; 14 Cardiovascular Medicine, University of Pennsylvania, Philadelphia (S.M.D.).

**Keywords:** actins, genotype, hypertrophic cardiomyopathy, myosin, sarcomere

## Abstract

Pathogenic variants in *MYBPC3*, encoding cardiac MyBP-C (myosin binding protein C), are the most common cause of familial hypertrophic cardiomyopathy. A large number of unique *MYBPC3* variants and relatively small genotyped hypertrophic cardiomyopathy cohorts have precluded detailed genotype-phenotype correlations.

**Methods:**

Patients with hypertrophic cardiomyopathy and *MYBPC3* variants were identified from the Sarcomeric Human Cardiomyopathy Registry. Variant types and locations were analyzed, morphological severity was assessed, and time-event analysis was performed (composite clinical outcome of sudden death, class III/IV heart failure, left ventricular assist device/transplant, atrial fibrillation). For selected missense variants falling in enriched domains, myofilament localization and degradation rates were measured in vitro.

**Results:**

Among 4756 genotyped patients with hypertrophic cardiomyopathy in Sarcomeric Human Cardiomyopathy Registry, 1316 patients were identified with adjudicated pathogenic truncating (N=234 unique variants, 1047 patients) or nontruncating (N=22 unique variants, 191 patients) variants in *MYBPC3*. Truncating variants were evenly dispersed throughout the gene, and hypertrophy severity and outcomes were not associated with variant location (grouped by 5′–3′ quartiles or by founder variant subgroup). Nontruncating pathogenic variants clustered in the C3, C6, and C10 domains (18 of 22, 82%, *P*<0.001 versus Genome Aggregation Database common variants) and were associated with similar hypertrophy severity and adverse event rates as observed with truncating variants. MyBP-C with variants in the C3, C6, and C10 domains was expressed in rat ventricular myocytes. C10 mutant MyBP-C failed to incorporate into myofilaments and degradation rates were accelerated by ≈90%, while C3 and C6 mutant MyBP-C incorporated normally with degradation rate similar to wild-type.

**Conclusions:**

Truncating variants account for 91% of *MYBPC3* pathogenic variants and cause similar clinical severity and outcomes regardless of location, consistent with locus-independent loss-of-function. Nontruncating *MYBPC3* pathogenic variants are regionally clustered, and a subset also cause loss of function through failure of myofilament incorporation and rapid degradation. Cardiac morphology and clinical outcomes are similar in patients with truncating versus nontruncating variants.

## Abstract 2

Supplemental Digital Content is available in the text.

Familial hypertrophic cardiomyopathy (HCM) is an autosomal dominant condition, and pathogenic variants in cardiac MyBP-C (myosin binding protein C; encoded by the gene, *MYBPC3*) are the most common cause.^1^ MyBP-C is a sarcomeric protein that binds both actin and myosin and regulates cardiac contractility by modulating myofilament sliding velocity.^2,3^ Because a large number of unique *MYBPC3* variants have been associated with HCM, small, single-center cohorts have had limited capacity to systematically analyze genotype-phenotype relationships, particularly given the marked variability in penetrance of *MYBPC3*-associated HCM.^4–7^ Resolving these gaps in knowledge will be critical to further personalized risk assessment and management of patients with HCM.

Most *MYBPC3* pathogenic variants are frameshift, nonsense, or splice-site variants that result in premature termination codons (PTCs). PTC-containing transcripts are targeted for degradation through nonsense mediated RNA decay, and hence may cause disease through allelic loss of function (resulting in reduced levels of MyBP-C). Consistent with allelic insufficiency, we and others have shown a ≈40% reduction in MyBP-C in heart tissue from patients with HCM,^8,9^ due to a rate-limiting reduction in *MYBPC3* mRNA.^10^ These studies support the hypothesis that truncating variants in *MYBPC3* likely exert a similar primary effect, independent of the specific variant locus. However, comparative analyses across the full genotypic and phenotypic spectrum of truncating variants have not been possible due to the small size of previously available cohorts. Distinct from truncating *MYBPC3* variants, nontruncating pathogenic variants (including missense and short in-frame deletions/insertion variants) account for ≈15% of *MYBPC3* HCM. The mechanism(s) of *MYBPC3* nontruncating pathogenic variants are largely unknown, and it is unclear whether phenotypic expression or clinical outcomes are different in patients carrying missense variants.^7,11^ A greater understanding of the disease-causing mechanism(s) of nontruncating *MYBPC3* pathogenic variants through functional analyses could improve adjudication of variant pathogenicity and expand the pool of clinically actionable gene test results.

Here, we use the largest registry of combined genetics and clinical data for HCM to date, the Sarcomeric Human Cardiomyopathy Registry^1^ (SHaRe), to generate an adjudicated and comprehensive compendium of *MYBPC3* variation, analyze regional variation within *MYBPC3*, and correlate clinical phenotypes. We find that pathogenic truncating variants are homogeneously distributed throughout the gene, in contrast to nontruncating *MYBPC3* pathogenic variants that cluster in specific protein domains. Disease severity is highly variable in *MYBPC3* HCM, and we show that this variability is largely independent of variant location or the specific truncating or nontruncating variant based on both disease severity metrics and clinical outcomes. Finally, we experimentally test functional effects of nontruncating pathogenic variants in the identified variant-enriched domains and identify a subset that exhibit allelic loss of function.

## Methods

The methods used are described for purposes of replicating the study procedure. Individual patient data will not be made available for purposes of reproducing the results. The study was independently approved by the institutional review board at each center. A detailed methods section is available in the Data Supplement.

## Results

### Clinical Profile of *MYBPC3* Mutation HCM

Among these patients with pathogenic *MYBPC3* variants, 1238 (94%) carried single pathogenic variants in *MYBPC3* without pathogenic variants in other sarcomere genes and comprised the primary study group. The largest subset of these patients (N=1047, 71%) carried truncating *MYBPC3* variants (234 unique variants), while 29% (N=191) had nontruncating variants (22 unique variants).

**Figure 1.**
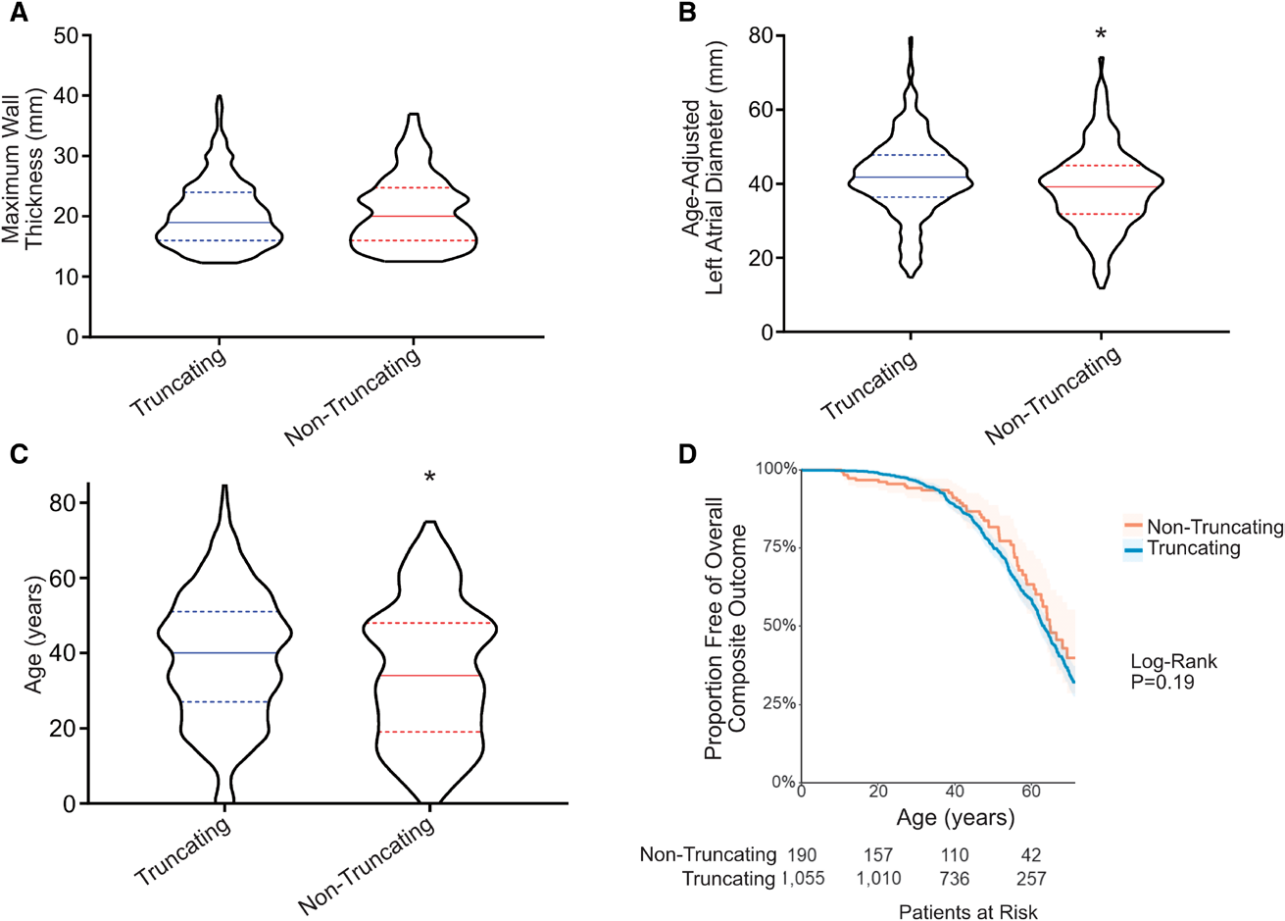
**MYBPC3 nontruncating pathogenic variants cause similar phenotypic severity and adverse event rates as truncating variants.** **A**, Distributions in maximum wall thickness demonstrate broad phenotypic variance and similarity between truncating and nontruncating *MYBPC3* pathogenic variant groups. Data is shown in violin plots with median and interquartile range. **B**, Average age-adjusted left atrial diameter was smaller among nontruncating pathogenic variant carriers. **C**, Broad variability in disease severity is reflected by range in age of diagnosis in both *MYBPC3* groups, with a modestly lower average age of diagnosis among nontruncating pathogenic variant carriers. **D**, Kaplan-Meier survival analysis shows no difference in the composite adverse event rate from time of birth between truncating and nontruncating pathogenic variant groups. Composite outcome consisted of first occurrence of any of the following: sudden cardiac death, resuscitated cardiac arrest, appropriate implantable cardioverter-defibrillator therapy, cardiac transplantation, left ventricle (LV) assist device implantation, LV ejection fraction <35%, or New York Heart Association class III/IV symptoms, atrial fibrillation (AF), stroke, or death.

The demographic and clinical profile of patients with *MYBPC3* pathogenic variants is shown in Table 1. The majority (76%) of patients presented in early-mid adulthood (age 18–60 years) with a minority of pediatric (13%) or late adulthood (10%) presentations. The average age of diagnosis was younger among patients with nontruncating pathogenic variants due to a greater percentage with pediatric diagnoses (24% versus 11%, *P*=0.0001). Maximum left ventricular (LV) wall thickness was greater in the relatively small subset of pediatric patients with nontruncating variants but was similar in other age groups. LV ejection fraction was similarly elevated at a young age in both groups and declined similarly in later age groups. Left atrial diameter progressively increased to a similar extent with increasing age in both truncating and nontruncating groups (with the single exception of the smaller sized group of nontruncating variant patients at age >60; N=17). The distributions of maximum wall thickness, left atrial size, and age of diagnosis among nontruncating and truncating pathogenic variant cases are shown in Figure 1A through 1C. Time to event analysis for composite adverse outcomes revealed no difference between patients with nontruncating or truncating pathogenic variants (Figure 1D), and this result was not different when including probands only (Figure IA in the Data Supplement). There were similarly no differences between the groups for heart failure or ventricular arrhythmia composite outcomes (not shown).

**Table 1.**
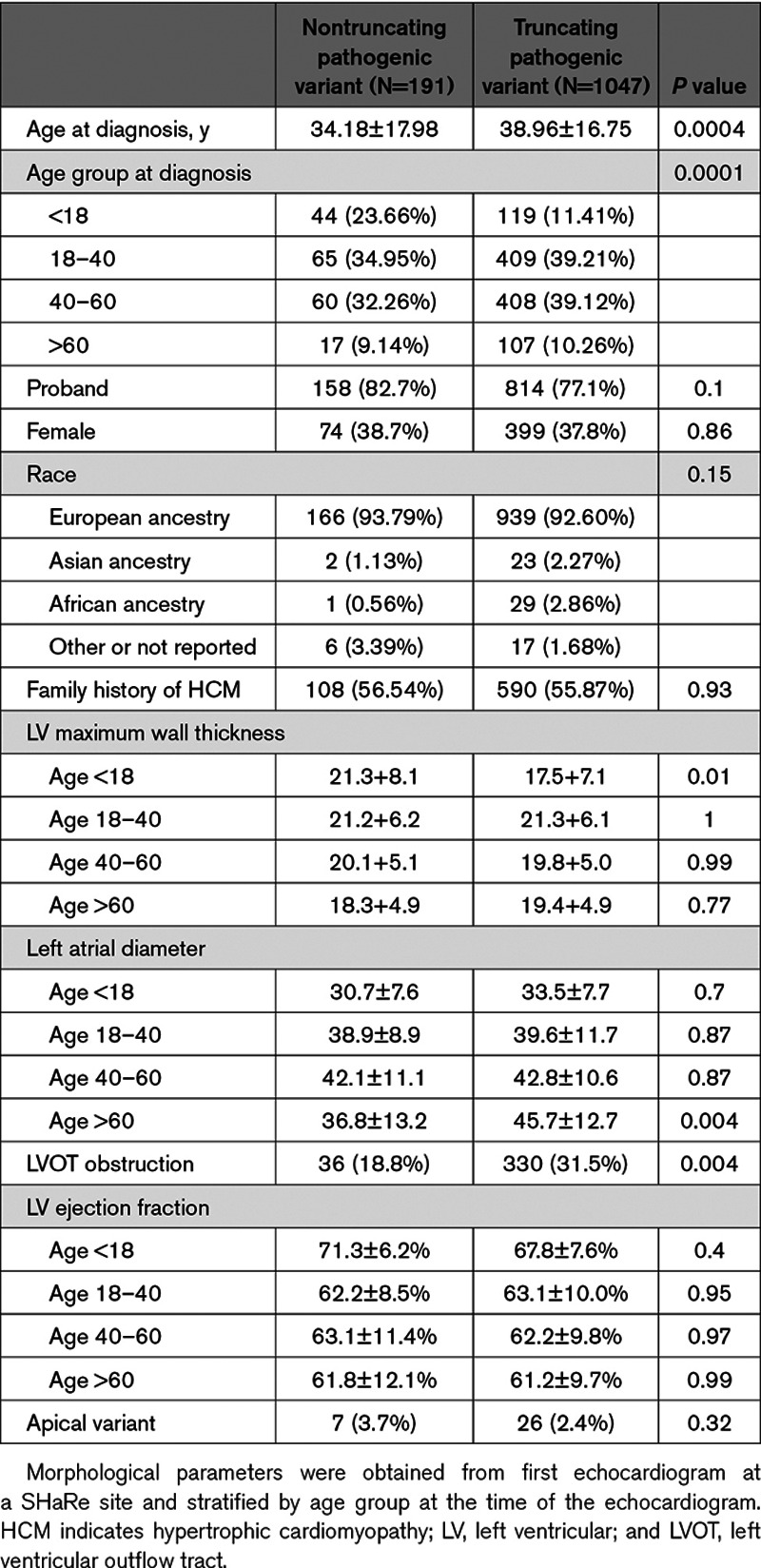
Demographic Characteristics of Patients With Truncating and Nontruncating *MYBC3* Pathogenic Variants

### Morphological Severity and Adverse Events Are Similar Across Truncating *MYBPC3* Pathogenic Variants

**Figure 2.**
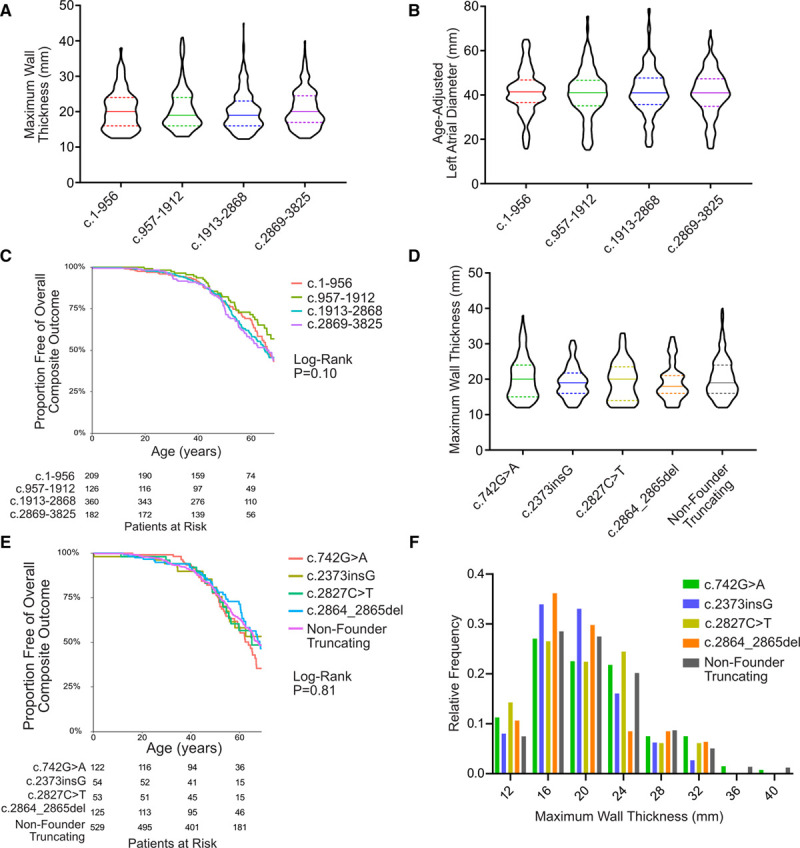
**MYBPC3 truncating pathogenic variants cause similar phenotypic severity regardless of variant locus or type.** **A** and **B**, Truncating *MYBPC3* variants were categorized by locus quartiles within the gene to examine whether N-terminal or C-terminal truncations exert different effect sizes. No difference in extent of hypertrophy (**A**) or left atrial diameter (**B**) are observed. **C** and **D**, Four founder populations within Sarcomeric Human Cardiomyopathy Registry (SHaRe) were compared with determine whether phenotypic severity is different in the setting of these 4 distinct truncating variant types (c.742G>A=exonic splice variant, c.2373insG=frameshift, c.2827C>T=nonsense, c.2864_2865del=frameshift). No difference was observed either in the variance/distribution of hypertrophy (**C**) or in the magnitude of hypertrophy (**D**).

If truncating *MYBPC3* variants cause allelic insufficiency as their primary consequence, then the location of the variant within the gene would not be expected to influence the disease severity. To test this, we categorized truncating *MYBPC3* pathogenic variants into quartiles by 5′ to 3′ location and compared morphological markers of severity and adverse outcomes. We found no statistically significant difference in maximum wall thickness or age-adjusted left atrial diameter among these groups (Figure 2A and 2B). Composite adverse events were also similar when stratified by variant location quartile (Figure 2C) or by truncating variant type (Figure IB in the Data Supplement).

### Morphological Severity, Adverse Events, and Variability in Phenotype Are Similar Among Founder and Nonfounder Truncating *MYBPC3* Pathogenic Variants

Several founder truncating pathogenic variants in *MYBPC3* have a high prevalence among patients with HCM. In SHaRe, 4 distinct founder truncating variants exist in large numbers, enabling comparison across subgroups that share the same primary causative sarcomere gene mutation. These founder populations consisted of 142 individuals with the c.742G>A variant (exonic splice variant causing exon skipping and PTC^12^), 143 with the c.2373insG variant (insertion variant causing frameshift and PTC),^13^ 67 with the c.2827C>T variant (nonsense variant), and 58 with the c.2864_2865del variant (deletion causing frameshift and PTC). LV hypertrophy was similar across each of these 4 founder populations and the remaining nonfounder truncating variant patients (N=638), further supporting that different truncating variants exert a similar effect (Figure 2D). Additionally, adverse events were similar in each founder population compared with patients with nonfounder truncating variants (Figure 2E).

HCM is known to have broad variance in phenotypic severity across individuals. This variance in expressivity has been thought to be due to heterogeneity of effect size of underlying pathogenic variants, the influence of background genetic variation (ie, genetic modifiers), and clinical comorbidities.^14–17^ Taking advantage of the founder populations, we compared variances across these subgroups each carrying identical pathogenic variants. As shown in the histogram plot of maximum wall thickness in Figure 2F, the 4 founder populations demonstrate similar variance (mean of SDs, 5.96±0.79 mm) compared with the remainder of the truncating variant population (SD, 5.98 mm; *P*=NS). Taken together, these findings indicate that truncating variants likely exert a similar primary effect, and the marked variance in disease phenotype among truncating variant patients is caused by additional genetic and nongenetic factors, independent of the driving *MYBP3* variant.

### Variant Classification and Distribution of Truncating and Nontruncating *MYBP3* Pathogenic Variants in HCM

*MYBPC3* truncating variant types in SHaRe patients consisted of 110 unique insertion/deletion variants, 55 unique nonsense variants, and 69 unique splice variants (Table I in the Data Supplement). Classification of potential splice variants is complicated by the fact that only a portion of splice consensus sites are strictly conserved. The 69 unique splice pathogenic variants were classified through application of the American College of Medical Genetics and Genomics Association for Molecular Pathology criteria, combined with enrichment in SHaRe, prior experimental confirmation, and independent experimental confirmation in select cases (Tables I and II and Figure II in the Data Supplement). These criteria left a total of 26 of 99 potential intronic variants classified at variant of unknown significance (VUS) status. Six exonic splice variants were identified at the last base pair position in their respective exons (donor -1 position), 4 of which have had prior experimental confirmation of splice disruption in human heart tissue.^12,18^ These splice variants (c.655G>C, c.772G>A, c.772G>C, c.1090G>A, c.1624G>C, c.1790G>A) were consequently classified as truncating—an important distinction since erroneous classification as missense variants would impact clustering analysis of the nontruncating variants. Comparison of our clinical-genetics assignment of variant pathogenicity to the *MYBPC3* splice variant prediction mini-gene splice assay developed by Ito et al^19^ demonstrated a high, though not perfect, level of concordance, with 20 out of 23 variants (87%) in agreement (Tables I and III in the Data Supplement). Nontruncating pathogenic variants were less common than truncating variants, with only 22 unique variants meeting criteria for pathogenicity, present in a total of 191 patients carrying a single sarcomere gene pathogenic variant (15% of *MYBPC3* pathogenic variant patients). The potential pathogenicity of 147 nontruncating VUS’s could not be resolved with clinical data from SHaRe.

**Figure 3.**
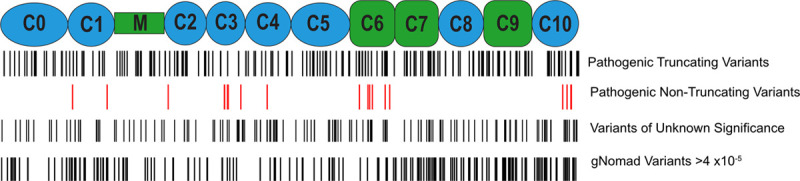
**Distribution of MYBPC3 pathogenic variants, variants of unknown significance, and common Genome Aggregation Database (gnomAD) variants relative to MyBP-C (myosin binding protein C) protein domains.** Truncating *MYBPC3* pathogenic variants are dispersed homogeneously throughout the gene, while nontruncating pathogenic variants exhibit clustering in the C3, C6, and C10 domains (18 of 22, 82%). Nontruncating variants of unknown significance are dispersed throughout the gene, as are gnomAD common variants (ie, allele frequency >4×10^−5^).

To determine regional variation in the distribution of *MYBPC3* pathogenic variants, we mapped all unique *MYBPC3* pathogenic variants in SHaRe by location within the coding sequence, stratified by truncating or nontruncating variant type (Figure 3). Truncating variants were dispersed throughout the coding regions of the gene without evidence of regional clustering. In addition, unique truncating variants were similarly prevalent in the N-terminus (including a variant that disrupts the start codon). In contrast, nontruncating pathogenic variants were primarily localized in the C3, C6, and C10 domains (18 of 22, 82%)—as compared with nontruncating common variants in the Genome Aggregation Database that were distributed throughout the gene (Figure 1, Tables III and IV in the Data Supplement). The C3 domain alone accounted for most individuals with *MYBPC3* nontruncating variants in SHaRe (177 of 191, 93%). Among Genome Aggregation Database common variants, a lower percentage (17%, 23 of 135) localized to the C3, C6, or C10 domains (*P*<0.0001 compared with SHaRe).

### Experimental Confirmation of Domain Specific Effects of MyBP-C Nontruncating Pathogenic Variants on Myofilament Incorporation and Degradation Rate

**Figure 4.**
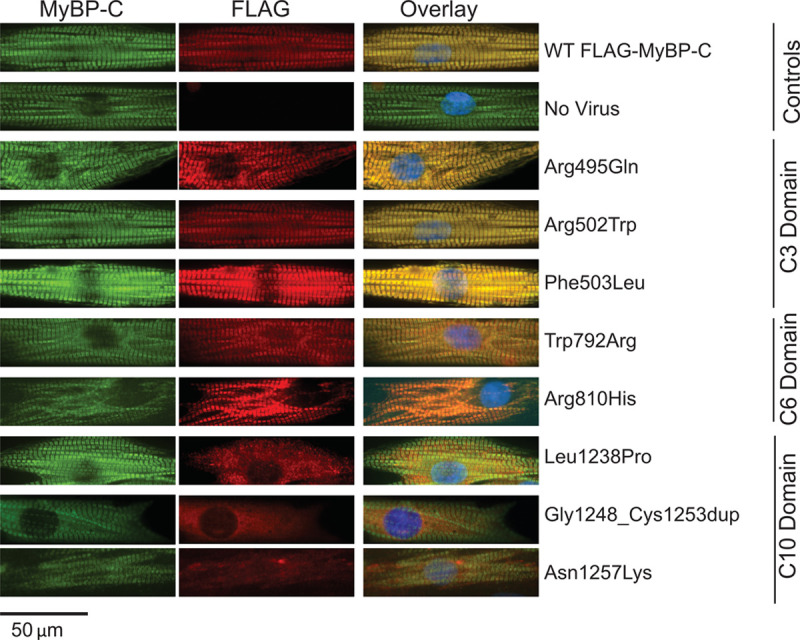
**Nontruncating MyBP-C (myosin binding protein C) mutant protein localizes to the myofilament for C3 and C6 domain mutants but does not incorporate into myofilaments for C10 domain mutants.** To determine whether mutant MyBP-C proteins integrate normally into the myofilaments, both FLAG-tagged control and mutant constructs were cloned into an adenoviral vector that was then used to transduce neonatal rat ventricular myocytes (NRVMs). Forty-eight hours following transduction, NRVMs were immunofluorescently labeled with an anti-MyBP-C antibody to detect both endogenous and exogenously expressed MyBP-C (**left** column) and an anti-FLAG antibody to detect only the transduced MyBP-C (**middle** column). This system achieved stable integration of FLAG-control MyBP-C into myofilaments (**top** row) with no FLAG signal detected without viral transduction (second row). Nontruncating mutant MyBP-C for C3 and C6 domain pathogenic variants exhibited normal myofilament integration while C10 mutant MyBP-C exhibited poor or no myofilament localization.

While strong evidence supports allelic insufficiency is the primary mechanism across the spectrum of truncating *MYBPC3* variants, the mechanism(s) of nontruncating *MYBPC3* pathogenic variants has not been resolved. We hypothesized that some nontruncating *MYBPC3* pathogenic variants may also cause loss of function, but through lack of normal protein localization or structural stability rather than reduced expression. Therefore, we first tested whether exogenously expressed MyBP-C with nontruncating pathogenic variants incorporates normally into the myofilaments. We expressed FLAG-epitope labeled MyBP-C with or without pathogenic nontruncating variants in neonatal rat ventricular myocytes and analyzed localization by immunofluorescence. We found that MyBP-C containing representative C3 or C6 domain nontruncating variants localized normally to the sarcomere A bands while MyBP-C containing C10 domain nontruncating variants was essentially absent from the myofilaments (Figure 4).

**Figure 5.**
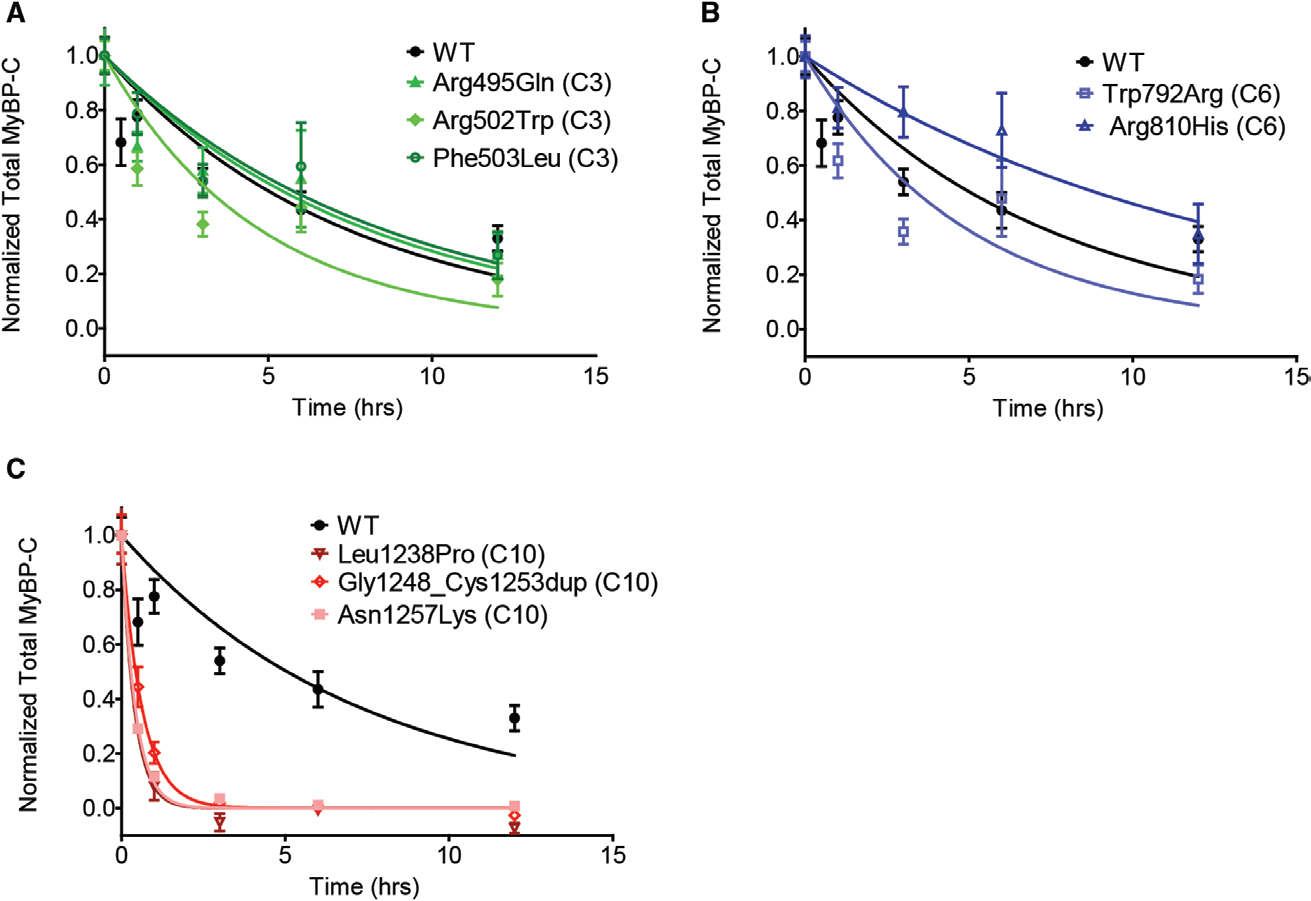
**Nontruncating mutant MyBP-C (myosin binding protein C) degradation rates measured by cyclohexamide pulse chase demonstrate rapid degradation for C10 domain nontruncating mutant MyBP-C.** To determine whether nontruncating *MYBPC3* pathogenic variants alter protein stability, neonatal rat ventricular myocytes were transduced with adenoviral constructs expressing wild-type (WT) control and nontruncating mutant MyBP-C. Cyclohexamide was administered at 0, 30 min, 1 h, 3 h, 6 h, and 12 h to inhibit protein synthesis and MyBP-C was measured (see Methods in the Data Supplement). Data from 2 or more independent experiments performed in quadruplicate were fit to a first order exponential decay curve. The same control data (from FLAG-labeled wild-type expressed MyBP-C) is depicted on each graph (**A–C**). **A** and **B**, C3 and C6 mutant MyBP-C demonstrates similar degradation rates as control. **C**, C10 mutant MyBP-C demonstrates rapid degradation compared with control. Data is represented as mean±SEM. The calculated half-lives with 95% CIs are shown in Table 2.

A lack of mutant MyBP-C myofilament incorporation could be either due to perturbation of binding sites required for correct localization or protein instability. To determine if pathogenic variants in the C10 domain result in protein destabilization, we performed cyclohexamide pulse-chase experiments using neonatal rat ventricular myocytes transduced with FLAG-tagged mutant MyBP-C for representative variants. Consistent with MyBP-C destabilization as a consequence of pathogenic variants in the C10 domain, we found a marked 90% reduction in protein half-life (Figure 5 and Table 2). In contrast, most pathogenic variants in the C3 and C6 domains resulted in MyBP-C protein half-lives that were not significantly different from wild-type MyBP-C, though the Arg502Trp variant resulted in a modest 36% shorter protein half-life compared with wild-type (*P*=0.04). Paradoxically, the Arg810His variant resulted in a 44% prolonged MyBP-C protein half-life compared with wild-type (*P*=0.008).

**Table 2.**
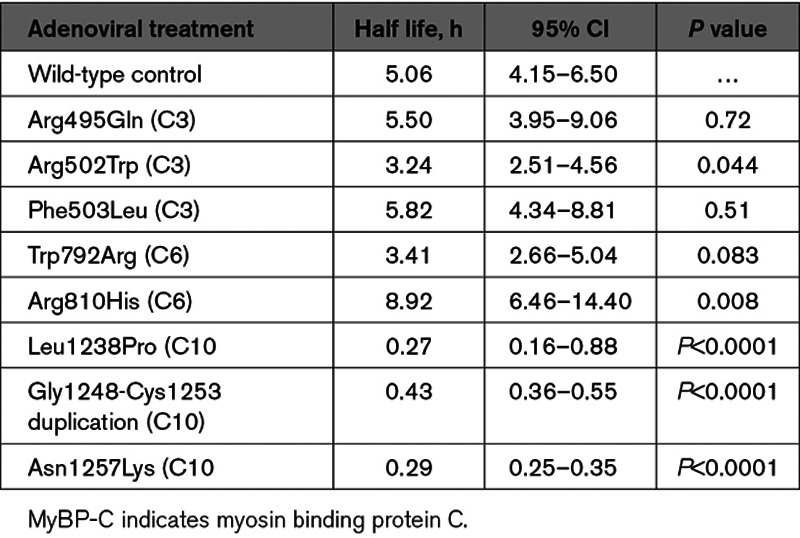
Nontruncating Mutant MyBP-C Degradation Rates Measured by Cyclohexamide Pulse Chase

## Discussion

Despite genetic variants in *MYBPC3* being the most common cause of familial HCM, identifying genotype-phenotype correlations has been elusive, due to the large number of individual pathogenic variants and small numbers of patients previously available to study from single centers. Here, we harness the largest cohort of genotyped patients with HCM to comprehensively describe *MYBPC3* genetic variation and associated clinical phenotypes.

A convergent theory of allelic insufficiency from truncating *MYBPC3* variants has emerged from human tissue, rodent, and induced pluripotent stem cell model systems.^8,10,20–22^ Reduction in MyBP-C relative to myosin alters sliding velocities as actin-myosin sliding reaches the C-zones, where MyBP-C is specifically present, resulting in a more rapid contractile deceleration toward peak force development.^2,8,10,23^ However, clinical-genetics data to confirm this theory have been notably absent. Our findings of a homogeneous distribution of HCM-causing truncating variants throughout *MYBPC3*, similar phenotypic severity across spatial quartiles in the coding sequence, and similar adverse event rates support the theory that disease results from a biologically similar loss of function mechanism across truncating variants, as opposed to dominant negative consequences from truncated MyBP-C protein (which has not been detectable in human heart or cellular models^9,10,12^). Furthermore, we found that 4 founder populations, with distinct truncating variants and sizable numbers in SHaRe, exhibit similar disease severity and adverse event rates as compared with nonfounder truncating variant patients. This result extends findings from a single site investigation of the Netherlands founder cohort,^24^ and counters smaller series that have suggested less pathogenic effects in truncating variant founder cohorts.^25,26^ A major implication of these results is that patients with truncating *MYBPC3* variants would likely derive similar benefit from targeted treatment approaches irrespective of the specific location of the truncating variant.

We further leveraged the truncating variant founder populations in SHaRe to investigate the variability in expressivity in HCM. HCM exhibits vast genetic and phenotypic heterogeneity, which has been a major challenge in determining genotype-phenotype relationships.^27^ We found that patients with founder variants had a similar distribution of phenotypic features and clinical outcomes as nonfounder patients with HCM with truncating variants. This finding suggests that the variability in disease phenotype among *MYBPC3* truncating variant carriers is not dictated solely by the primary pathogenic variant. An important implication of this finding is that additional genetic and nongenetic modifiers likely account for the broad variance in phenotypic severity among patients with *MYBPC3* HCM.

We also demonstrate that *MYBPC3* nontruncating pathogenic variants, accounting for 15% of *MYBPC3* pathogenic variants, generally had a similar phenotypic effect as truncating variants. Minor differences between the groups included a modestly greater proportion of pediatric diagnoses in the nontruncating group and modestly reduced prevalence of LV outflow tract obstruction. However, maximal LV wall thickness across all other age groups and adverse event rates were highly similar.

Because nontruncating variants are robustly adjudicated in SHaRe, we were able to identify strong evidence of domain clustering. We then demonstrated that a subgroup of nontruncating pathogenic variants (those in the C10 domain) renders the resultant mutant protein susceptible to rapid degradation, resulting in a loss of function mechanism similar to truncating variants. In contrast, we show no destabilization in the majority of C3 and C6 domain mutant proteins, which integrate normally in myofilaments. The C3 variant Arg502Trp alters the electrostatic properties of the domain, but how this alteration affects MyBP-C function is not known.^28^ In engineered heart tissue, overexpression of the C3 mutant Gly531Arg (not present in SHaRe), caused hypercontractility at low calcium levels and was not able to rescue MyBP-C knock-out tissues.^29^ Further study is required to fully elucidate the impact of C3 and C6 pathogenic variants on contractile function.

In contrast to the clustering evident for pathogenic nontruncating variants, VUS’s in *MYBPC3* were relatively common in the SHaRe cohort (N=148, 87% of all unique *MYBPC3* nontruncating variants). Accurate prediction of pathogenicity of sarcomere VUS’s is a major challenge for interpretation of genetic testing results and determination of the suitability for cascade testing in family members. Although we confirmed enrichment of nontruncating pathogenic variants in specific MyBP-C domains, as also shown in an independent cohort by Walsh et al,^30^ the presence of common variants in Genome Aggregation Database in these same domains should preclude a complete reliance on a generalized domain-centric approach to determine variant pathogenicity. Nevertheless, the presence of a variant in the C3, C6, or C10 domains in a patient with HCM increases the probability of pathogenicity and could be used as a supportive criterion with other clinical variables in variant classification. Moreover, identifying VUS’s that cause protein instability could be a useful strategy for functional annotation of variants.

Several limitations to our study should be considered. This was a retrospective, observational study. Although we analyzed by far the largest cohort of patients with HCM with *MYBPC3* pathogenic variants to date, the study may be underpowered to detect small differences in phenotype severity or adverse events between groups. In addition, we analyzed pathogenic variant carriers in groups based on variant type and location, but further subdivision to individual pathogenic variants was only feasible for the founder subpopulations. As such, differences in effect size for specific pathogenic variants, particularly in the case of the nontruncating variants, could still exist. Both the SHaRe population and Genome Aggregation Database populations predominantly consist of individuals from European ancestry. Although these attributes lend confidence to the calculation of the odds ratios for HCM-associated versus common population variants reported here, the results are not necessarily representative of genetic variation in other ancestries. Relatedly, the SHaRe population has a greater proportion of patients with HCM with truncating founder variants due to inclusion of certain European sites (the Netherlands, Italy). Lastly, we strategically focused experimental testing of nontruncating pathogenic variants to the impact on protein stability and only examined a subset of representative variants. Future work will be needed to further resolve the functional effects of pathogenic nontruncating *MYBPC3* variants that do not destabilize the protein structure and extending these analyses more comprehensively across *MYBPC3* nontruncating variants.

In conclusion, we leverage the largest cohort of patients with *MYBPC3* pathogenic variants to date to develop a compendium of benign, pathogenic, and uncertain *MYBPC3* variants and identify genotype-phenotype correlations. Our results demonstrate that phenotypic severity and clinical outcomes are similar across the range of *MYBPC3* pathogenic variant carriers, without obvious associations based on the location of truncating variants, founder, or nonfounder truncating variant carriers, or truncating versus nontruncating variants. These findings highlight the need to identify additional background genetic and nongenetic modifiers that influence the broadly variable HCM disease phenotype. In addition, we show that nontruncating pathogenic variants cluster in particular MyBP-C domains, with those variants in the C10 domain exhibiting protein destabilization leading to loss of function, in contrast to a second subset exhibiting normal myofilament incorporation and stability.

## Sources of Funding

Funding for SHaRe has been provided through an unrestricted research grant from Myokardia, Inc, a startup company that is developing therapeutics that target the sarcomere. MyoKardia, Inc, had no role in approving the content of this article. Dr Helms is supported by funding from the National Institutes of Health (K08HL130455). Dr Thompson is supported by the National Institutes of Health (T32 HL007853). Dr Ware is supported by the Wellcome Trust (107469/Z/15/Z) and the Medical Research Council (United Kingdom). Dr Ingles a recipient of a National Health and Medical Research Council (NHMRC) Career Development Fellowship (No. 1162929). Dr Semsarian is the recipient of a NHMRC Practitioner Fellowship (No. 1059156). Dr Olivotto is supported by the Italian Ministry of Health (“Left Ventricular Hypertrophy in Aortic Valve Disease and Hypertrophic Cardiomyopathy: Genetic Basis, Biophysical Correlates and Viral Therapy Models” [RF-2013-02356787] and NET-2011-02347173 [Mechanisms and Treatment of Coronary Microvascular Dysfunction in Patients sith Genetic or Secondary Left Ventricular Hypertrophy]) and by the Tuscany Registry of Sudden Cardiac Death (ToRSADE) project (FAS-Salute 2014, Regione Toscana). Dr Ho is supported by funding from the National Institutes of Health (1P50HL112349 and 1U01HL117006). Dr Day is supported by funding from the National Institutes of Health (R01 11572784), the American Heart Association (grant in aid), the Children’s Cardiomyopathy Foundation, and the Protein Folding Disease Initiative (University of Michigan).

## Disclosures

Dr Helms, Dr Ho, Dr Day, Dr Saberi, Dr Olivotto, Dr Colan, Dr Ingles, and E.A. Ashley receive research support from MyoKardia, Inc. The other authors report no conflicts.

## Supplementary Material


