# Supplementary material for: Spatial and Functional Distribution of MYBPC3 Pathogenic Variants and Clinical Outcomes in Patients With Hypertrophic Cardiomyopathy
Source: Circ Genom Precis Med. 2020 Aug 25;13(5):396–405. doi: 10.1161/CIRCGEN.120.002929 (PMC7676622; doi:10.1161/CIRCGEN.120.002929)
Supplement: Supplementary file 1 [file hcg-13-396-s001.pdf]

Supplemental Material

Expanded Methods

SHaRe Registry Data Extraction and *MYBPC3* Variant Classifications

The generation of the centralized SHaRe database has been previously described.[1] De-identified data from each participating site is updated on a quarterly basis to a secure centralized database (Boston Advanced Analytics, Boston, MA). Data for this study were exported from quarter 1 of 2019. Institutional review board and ethics approval was obtained in accordance with policies applicable to each SHaRe site. Inclusion criteria included a site-designated diagnosis of HCM, as previously defined.[1] Only patients with a clinical diagnosis of HCM were included in the study (phenotype-negative family members were not included).

Clinically-indicated genetic testing was performed from each site. Variants were classified as pathogenic, likely pathogenic, variant of unknown significance (VUS), likely benign, or benign in accordance with American College of Medical Genetics and Genomics and Association for Molecular Pathology (ACMG/AMP) standards[2], largely as modified for *MYH7* cardiomyopathy.[3] Pathogenic and likely pathogenic variants were coalesced and are referred to as “pathogenic” for brevity. Each *MYBPC3* genetic variant was also independently reviewed by a subset of SHaRe investigators for this study. Criteria for pathogenicity included a combination of variant frequency in reported cases (including SHaRe), variant frequency in the Genome Aggregation Database (gnomAD)[4], evidence of segregation within families in SHaRe, and/or published. All rare frameshift and nonsense variants were considered pathogenic, and splice site variants were considered pathogenic if they affected critically conserved splice consensus sites (i.e. acceptor -1 or -2; donor +1 or +2) and/or if experimental evidence from human heart tissue showed aberrant splicing.[5, 6] All frameshift, nonsense, and splice site variants were designated as “truncating” for analysis. In-frame deletion or duplication variants that do not introduce PTCs (and hence are non-truncating), were categorized along with missense variants and considered as a

single group. All potential exonic splice variants were identified among missense variants. Exonic variants at the donor -1 position were considered as truncating; otherwise potential exonic variants were collated with missense variants and annotated as having a possible splice impact. Both intronic and exonic possible splice variants were cross-referenced to a recent *in silico* and mini-gene assay analysis of *MYBPC3* variants predicted to impact splicing.[7] We calculated odds ratios for variant alleles in SHaRe relative to gnomAD (i.e. proportion of variant alleles in SHaRe / proportion of variant alleles in gnomAD), consistent with the "PS4" rule from the modified ACMG/AMP standards, as supportive evidence.[3] For variants present in SHaRe but absent in gnomAD, a conservative upper bound population allele frequency of 4.5E-06 (i.e. 1/224,000) was used for the odds ratio calculation (given absence in a mean of 224,000 alleles sequenced in gnomAD). Variants were considered "likely benign" if the population allele frequency exceeded 0.004 and the odds ratio was <10-fold over the gnomAD allele frequency.

Comparison of SHaRe *MYBPC3* Variant Regional Distribution with Population Variants

To compare SHaRe variants to common population variation in *MYBPC3*, all *MYBPC3* variants and allele frequencies present in gnomAD were extracted. Since variants in sarcomere genes with allele frequencies >4E-05 in gnomAD are unlikely to be independently pathogenic for hypertrophic cardiomyopathy[8], gnomAD missense variants were filtered for variants exceeding this frequency. These relatively common population variants were then plotted in relation to SHaRe pathogenic variants to examine the distribution of tolerated variation versus pathogenic variation. Apparent clustering of non-truncating variants was then assessed by MyBP-C protein domain and compared to the proportions of variants in gnomAD using the Fisher exact test.

Clinical Metrics and Outcomes Analysis

For clinical severity and outcomes analyses, only individuals with single *MYBPC3* pathogenic or likely pathogenic variants were included. Clinical metrics of disease severity were obtained from echocardiography. Specifically, maximum LV wall thickness and left atrial diameter were selected for

quantification of disease severity since these measurements are: 1) available on nearly all patients in SHaRe, 2) continuous variables with approximately normal distributions, 3) indicative of disease severity in large cohorts.[9, 10] Since left atrial size increases with age, age-adjusted left atrial diameter was used to compare across groups. The atrial size was adjusted based on the linear rate of progression of atrial size by year observed among patients with sarcomeric HCM in SHaRe. A composite outcome was used to assess adverse events, consisting of first occurrence of any of the following: sudden cardiac death, resuscitated cardiac arrest, appropriate implantable cardioverter-defibrillator therapy, cardiac transplantation, LV assist device implantation, LV ejection fraction <35%, or New York Heart Association class III/IV symptoms, atrial fibrillation (AF), stroke, or death.

Isolation and Culture of Neonatal Rat Ventricular Myocytes

Neonatal rat ventricular myocytes (NRVMs) studies were approved by the University of Michigan Animal Care and Use Committee. NRVMs were isolated as previously described.[11] Briefly, myocytes were isolated from excised ventricles of 1- to 3-day-old SpragueDawley rats (Charles River) according to a modified version of the Worthington Neonatal Cardiomyocyte Isolation System (Worthington Biochemical Corporation). Ventricles were minced in ice-cold HBSS and predigested in 1 mg/ml trypsin (Worthington) at 4°C for 6 hours. Tissue was then digested in 30 U/ml purified collagenase (Worthington), dissolved in Media 199 (Invitrogen) with Earle's salts, L-glutamine, 2.2 g/l sodium bicarbonate, 2% penicillin/streptomycin, 25 mM HEPES, and 15% heat-inactivated qualified FBS (Invitrogen), in a Celstir 50-ml jacketed spinner flask (Wheaton) for 45 minutes at 37°C. Digested tissue was triturated and filtered through a 70-µm strainer, and then incubated for 20 minutes at room temperature to further digest partially degraded collagen. Cell suspensions were pre-plated on untreated plastic dishes for 1 hour at 37°C to reduce adherent fibroblast contamination, and then filtered through a 40-µm strainer. NRVMs were seeded on plates coated with 5 µg/ml bovine fibronectin (Sigma-Aldrich) or on fibronectin micropatterned PDMS coverslips in maintenance media (Media 199

with Earle's salts, L-glutamine, 2.2 g/l sodium bicarbonate, 2% penicillin/streptomycin, 25 mM HEPES, and 5% FBS). Micropatterned PDMS coverslips were used for myofilament localization studies since routine culture of NRVMs results in disordered myofilament organization, hindering precise analysis of sarcomere structures, while micropatterning reproducibly induces anisotropic cell growth and myofibrillar alignment.[12] Micropatterned PDMS stamps with 20-µm-wide rows spaced 3-µm apart were fabricated as previously described.[11]

Expression of FLAG-Tagged MyBP-C Constructs

MYBPC3 non-truncating mutants were generated by site-directed mutagenesis using the QuikChange II XL Kit (Agilent) from WT human *MYBPC3* cDNA. Adenovirus was generated with the ViraPower Adenoviral Gateway Expression Kit (Invitrogen) using the pAd/CMV/V5-DEST Gateway vector and amplified in HEK293A cells. A multiplicity of infection (MOI) of 10 for NRVM transduction was utilized for cyclohexamide chase assays and MOI 5 was utilized for immunofluorescence analysis. Untreated samples were used as negative controls throughout. For modeling of C3, C6, and C10 domain mutations, we selected the same mutations as for the computational modeling.

Immunofluorescence Analysis of MyBPC-C Myofilament Localization in NRVMs

NRVMs were plated on micropatterned PDMS at a density of 1.5 x10^5 cells/well in 6-well plates prior to viral transduction and incubated for 24 hours. Cells were transduced at MOI 5 with WT or mutant FLAG-MyBP-C adenovirus for 48 hours prior to paraformaldehyde fixation. Cells were permeabilized in TBS-T with 0.2% Triton X-100 (Sigma-Aldrich) for 8 minutes then blocked in 5% goat serum (Vector Biolabs) and 1 mg/ml bovine serum albumin (Sigma Aldrich) in TBS for 40 minutes. Antibody and staining conditions were as follows: MyBP-C, rabbit polyclonal 1:2,000 (kind gift from Samantha Harris, University of Arizona); FLAG M2, mouse monoclonal 1:200 (SigmaAldrich, F1804); goat anti-mouse IgG Alexa Fluor 594, 1:1,00 (ThermoFisher Scientific, A11005); and goat anti-rabbit IgG Alexa Fluor 488, 1:1,00 (ThermoFisher Scientific, A11008). PDMS substrates were mounted onto slides face-

up, ProLong Diamond Antifade (ThermoFisher Scientific) added, then topped with glass coverslips. Images were taken using Nikon Eclipse Ti. All viral constructs (WT and mutant FLAG-MYBPC3) were tested in at least duplicate in two separate NRVMs preparations. An untreated control cell was used to control for non-specific binding and autofluorescence.

Cyclohexamide Pulse-Chase Quantification of MyBP-C Degradation Rate

Unpatterned NRVMs were plated at a density of $4.0x10^4$ cells/well in 96-well culture plates and incubated for 24 hours prior to transduction at MOI 10 with WT or mutant MYBPC3 adenovirus for 48 hours. Maintenance media with and without 300 ug/ml cyclohexamide (Sigma-Aldrich) was prepared and cells were cultured in maintenance media without cyclohexamide until the appropriate time point. cyclohexamide treatment of 0, 30 minutes, 1 hour, 3 hours, 6 hours, and 12 hours was performed in quadruplicate wells. Four wells not transduced with virus were utilized as negative controls within each 96-well plate. Upon completion of the experiment, samples were lysed using 50µl/well alpha-LISA lysis buffer (Perkin Elmer, AL003C) and incubated at room temperature for 1 hour. Samples were stored at -80 Celsius sealed with an adhesive cover (microAmp optical adhesive film, Applied BioSystems).

MyBP-C was then quantified from the cyclohexamide pulse chase samples using the AlphaLISA® assay (Perkin Elmer). The AlphaLISA® is a bead based assay which uses luminescent oxygen-channeling chemistry. The analyte, (FLAG-MyBP-C) was captured by a mouse monoclonal FLAG antibody (SigmaAldrich, F1804) bound to an anti-mouse IgG donor bead (Perkin Elmer, AS104D) and a rabbit monoclonal MyBP-C antibody (kind gift from Samantha Harris, University of Arizona) bound to an anti-rabbit IgG acceptor bead (Perkin Elmer, AL104C). Laser irradiation of donor beads at 680 nm generates a flow of singlet oxygen, triggering a cascade of chemical events in nearby acceptor beads, which results in a chemiluminescent emission at 615 nm. In our assay 1 µl of WT or mutant MYBPC3 lysate was diluted to 5µl within alpha-LISA lysis buffer (Perkin Elmer, AL003C) and incubated with 10 µl of FLAG (1:1,000) and MyBP-C (1:1,000) antibodies in alpha-LISA assay buffer for 1 hour within ½ area 96 well white

bottom plates (Perkin Elmer, 6002290). Next 10 µl of donor (1:100) and acceptor (1:100) beads in Alpha-LISA assay buffer were added to each well and samples were incubated under foil, seal protected from light for 48 hours. Samples were read using the Perkin Elmer EnVision Multimode Plate Reader. Lysate from cells which did not undergo adenoviral treatment was used as a negative control. Given lower levels of total FLAG-MyBP-C for C10 mutants, 5 µl of lysate from cells treated with these viral constructs and untreated controls were utilized to remain in the dynamic range of the assay. 2- fold serial dilution of cellular lysates was performed as a positive control to ensure experimental samples remained within the dynamic range of the assay.

To analyze the cyclohexamide pulse chase data, the background (defined as average signal from the four wells untreated with adenovirus in each plate) was first subtracted from the raw signal. For each condition the time 0 data were normalized to 1 to yield a curve for the relative abundance of FLAG-MyBP-C over time. Data from two or more independent experiments performed in quadruplicate were fit to a first order exponential decay curve $[MyBP\text{-}C] = [MyBP\text{-}C]_{t=0}*e^{-kt}$ (with $[MyBP\text{-}C]_{t=0}$ normalized to 1), from which reaction constants k and half-lives $t_{1/2}$ calculated as $t_{1/2} = \ln(2)/k$ were determined.

Evaluation of Splice Variants

Human heart Tissue Procurement: Myocardial tissue was obtained from interventricular septum of subjects with HCM at time of surgical myectomy (N = 7). Intraventricular septal tissue was obtained from a gift of life donor hearts at time of explant (N = 1) to serve as a control. The donor heart was perfused with cardioplegia solution before removal. All tissues were snap-frozen in liquid nitrogen immediately after excision. The study was approved by the University of Michigan Institutional Review Board and subjects gave informed consent.

Sequencing of *MYBPC3* Splice Site Mutations: Total mRNA was extracted from human tissue using the Qiagen Fibrous Tissue Mini Kit (Cat. No. 74704) according to the manufactures protocol with the following adjustments. The ratio of buffer RLT: RNAse-free water was 600 µL: 290 uL, and 15 µl of

DNAse was added at time of extraction. Reverse transcription was performed on 1 µg of total RNA using the Omniscript RT kit (Qiagen) according to manufacturer's instructions. RNA and cDNA concentrations were determined and the purity verified using Nanodrop One (Thermo Scientific). When designated genomic DNA extraction was performed using DNEasy blood tissue kit (Qiagen) according to manufacturer's instructions.

PCR Amplification of Splice Sites: cDNA was PCR-amplified using primers designed using the NCBI primer blast tool and the SnapGene Viewer Application to detect exon exclusion and intron inclusion (Supplemental Tables 5-6). The PCR reaction was performed using the ThermoFisher DreamTaq Hot Start Green PCR Master Mix and the respective primers for each patient sample (see Supplemental Tables 5-6 for primer lists). The PCR product was separated using gel electrophoresis on 1-2% agarose gel. Individual bands were extracted and Sanger sequenced and compared to NCBI DNA and mRNA reference sequence for *MYBPC3*.

Statistical Analysis

Clinical data are presented as mean ± standard deviation and were analyzed by t-test for two groups or by ANOVA for >2 groups with Dunn's post hoc test for multiple comparisons. Composite outcomes were analyzed by the Kaplan-Meier method from time of birth. Analysis from time of birth is appropriate given that the genetic variant is present from birth and variability in time to, and reason for, clinical presentation could confound the results. Patients who did not have the outcome of interest were censored at the time of their last recorded follow-up in SHaRe. Patients with missing data on the occurrence or timing of events were not included in analyses of those outcomes. Statistical analyses were performed using GraphPad Prism software. P-values of <0.05 were considered statistically significant.

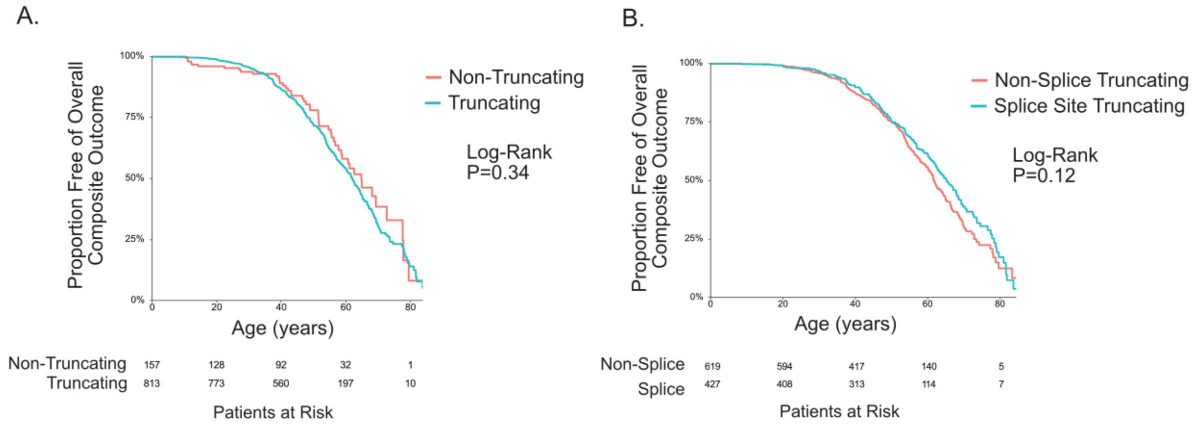

Supplemental Figure 1. **A.** Proband-only time to event analysis for the composite outcome showed no difference between truncating and non-truncating pathogenic variants. **B.** Comparison between non-splice site truncating (i.e. frameshift and nonsense) and splice site truncating variants showed no difference in the composite outcome.

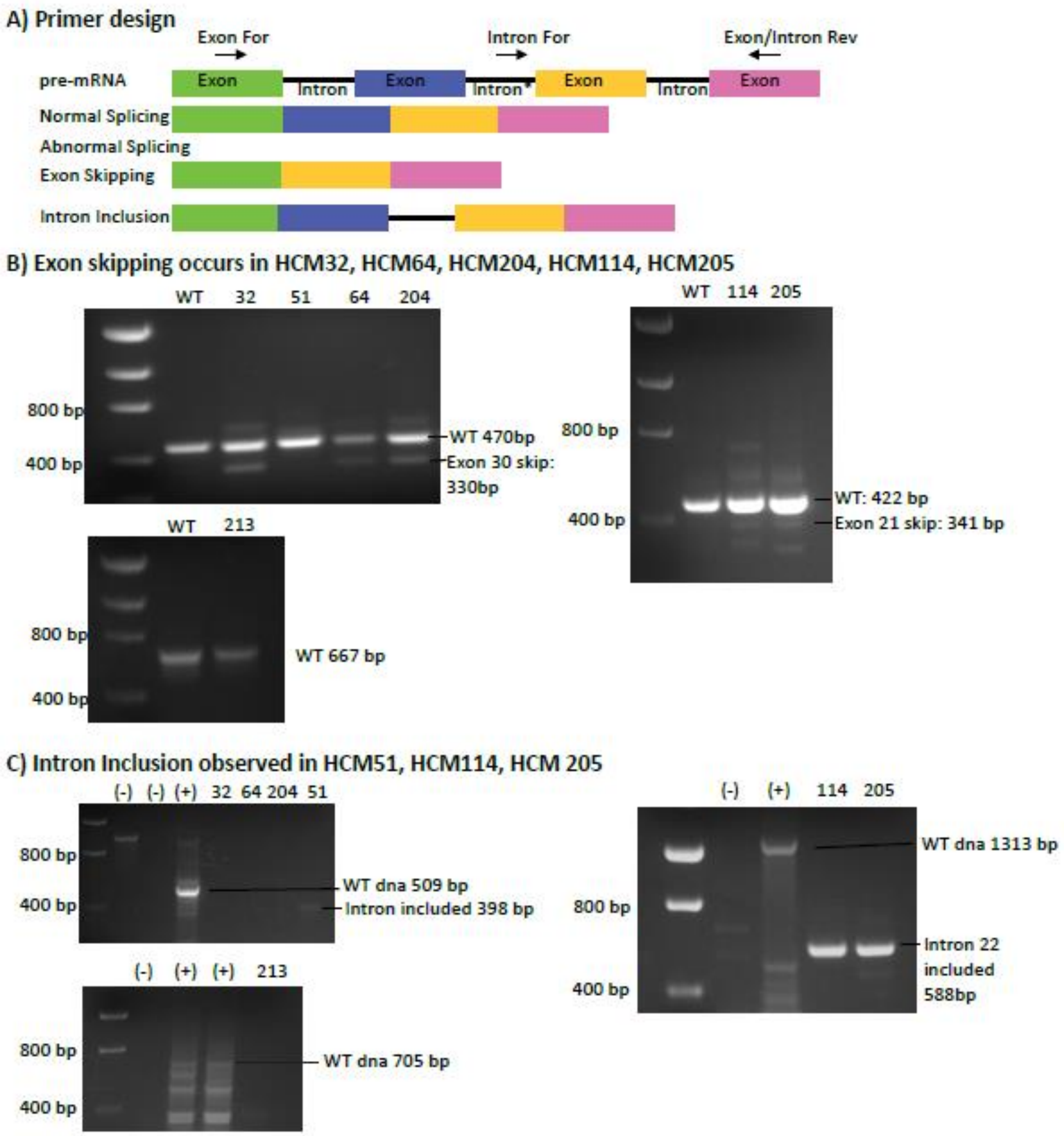

Supplemental Figure 2. Patient myectomy samples carying splice mutations were evaluated by PCR. A. Two sets of primers were designed for each intron mutation of interest – one that would detect exon skipping and one that would detect abnormal intron inclusion. PCR was performed in duplicate. **B.** Representative DNA gel and sequencing results are shown and demonstrate that exon skipping occured in samples HCM32, 64, 204, 114, 205. **C.** Intron inclusion is observed in samples HCM51, 114, 205 (no bands expected for WT mRNA). To demonstrate lack of DNA contamination during RNA extraction (which would result in a false positive for intron inclusion), a negative control sample from wild-type donor heart tissue was used (-) and genomic DNA was also extracted from this tissue (+). Primers were designed to span intron of interest and the following intron. Confirmatory sequencing results are summarized in Supplemental Table 2.

MYBPC3 Insertion/Deletion and Nonsense Mutations in SHaRe									
Nucleotide Variant	Amino Acid Variant	Truncating Mutation Type	Human Heart Abnormal Splicing Proven	Class	SHaRe Number of Cases	SHaRe Allele Frequency	gnomAD Allele Frequency	Odds Ratio (SHaRe Enrichment)	Ito, et al.[7], Positive Mini-Gene Assay
c.3G>C	p.Met1?	Frameshift	NA	P/LP	2	4.21E-04	4.33E-06	48.6	Yes
c.65_66insG	p.Ala23Argfs*26	Frameshift	NA	P/LP	1	2.10E-04		>23.5	
c.95del	p.Glu32Glyfs*7	Frameshift	NA	P/LP	1	2.10E-04		>23.5	
c.126G>A	p.Trp42*	Nonsense	NA	P/LP	1	2.10E-04		>23.5	
c.162del	p.Lys54Asnfs*13	Frameshift	NA	P/LP	1	2.10E-04		>23.5	
c.177_187del	p.Glu60fs*49	Frameshift	NA	P/LP	6	1.26E-03		>141.3	
c.208del	p.Glu70fs*25	Frameshift	NA	P/LP	1	2.10E-04		>23.5	
c.227_228insA	p.Ser78Ilefs*35	Frameshift	NA	P/LP	1	2.10E-04		>23.5	
c.237C>G	p.Tyr79*	Nonsense	NA	P/LP	1	2.10E-04		>23.5	
c.292G>C	p.Glu98*	Nonsense	NA	P/LP	1	2.10E-04		>23.5	
c.306_306del	p.Met103Cysfs*56	Frameshift	NA	P/LP	1	2.10E-04		>23.5	
c.362del	p.Pro121Argfs*38	Frameshift	NA	P/LP	2	4.21E-04		>47.1	
c.410C>G	p.Ser137*	Nonsense	NA	P/LP	1	2.10E-04		>23.5	
c.436_437insA	p.Thr146fs	Frameshift	NA	P/LP	1	2.10E-04		>23.5	
c.459del	p.Ile154Leufs*5	Frameshift	NA	P/LP	4	8.41E-04		>94.2	
c.484C>T	p.Gln162*	Nonsense	NA	P/LP	1	2.10E-04		>23.5	
c.540_559del	p.Ala181Cysfs*53	Frameshift	NA	P/LP	1	2.10E-04		>23.5	
c.551dup	p.Lys185Glufs*56	Frameshift	NA	P/LP	2	4.21E-04		>47.1	
c.553A>T	p.Lys185*	Nonsense	NA	P/LP	1	2.10E-04		>23.5	
c.613C>T	p.Gln205*	Nonsense	NA	P/LP	1	2.10E-04		>23.5	
c.676_701dup26	p.Gly235fs	Frameshift	NA	P/LP	1	2.10E-04		>23.5	
c.688del	p.Gln230fs	Frameshift	NA	P/LP	7	1.47E-03		>164.8	
c.791insG		Frameshift	NA	P/LP	1	2.10E-04		>23.5	
c.806C>A	p.Ser269*	Nonsense	NA	P/LP	1	2.10E-04		>23.5	
c.840_851+5del17		Frameshift	NA	P/LP	1	2.10E-04		>23.5	
c.897del	p.Lys301fs	Frameshift	NA	P/LP	4	8.41E-04		>94.2	
c.913_914del	p.Phe305Profs*27	Frameshift	NA	P/LP	19	3.99E-03		>447.4	
c.932C>A	p.Ser311*	Nonsense	NA	P/LP	4	8.41E-04		>94.2	
c.989del	p.Pro330Hisfs*20	Frameshift	NA	P/LP	1	2.10E-04		>23.5	
c.993dup	p.Glu332*	Nonsense	NA	P/LP	1	2.10E-04		>23.5	
c.1000G>T	p.Glu334*	Nonsense	NA	P/LP	3	6.31E-04		>70.6	
c.1015C>T	p.Gln339*	Nonsense	NA	P/LP	1	2.10E-04		>23.5	
c.1020C>G	p.Tyr340*	Nonsense	NA	P/LP	6	1.26E-03		>141.3	
c.1053_1054delinsTT	p.Arg351fs	Frameshift	NA	P/LP	2	4.21E-04		>47.1	

c.1120C>T	p.Gln374*	Nonsense	NA	P/LP	1	2.10E-04		>23.5	
c.1153_1168del	p.Val385Metfs*16	Frameshift	NA	P/LP	3	6.31E-04		>70.6	
c.1168del	p.His390Metfs*16	Frameshift	NA	P/LP	1	2.10E-04		>23.5	
c.1171del	p.Asp391Thrfs*15	Frameshift	NA	P/LP	1	2.10E-04		>23.5	
c.1174del	p.Ala392Leufs*14	Frameshift	NA	P/LP	15	3.15E-03		>353.2	
c.1201C>T	p.Gln401*	Nonsense	NA	P/LP	1	2.10E-04		>23.5	
c.1210C>T	p.Gln404*	Nonsense	NA	P/LP	7	1.47E-03		>164.8	
c.1235_1236del	p.Phe412*	Nonsense	NA	P/LP	3	6.31E-04		>70.6	
c.1354_1355insC	p.Val454Cysfs*21	Frameshift	NA	P/LP	1	2.10E-04		>23.5	
c.1357_1358DEL	p.Pro453Cysfs*21	Frameshift	NA	P/LP	2	4.21E-04		>47.1	
c.1359del	p.Val454Cysfs*12	Frameshift	NA	P/LP	3	6.31E-04		>70.6	
c.1377del	p.Leu460Trpfs*6	Frameshift	NA	P/LP	1	2.10E-04		>23.5	
c.1404del	p.Gln469fs	Frameshift	NA	P/LP	1	2.10E-04		>23.5	
c.1405C>T	p.Gln469*	Nonsense	NA	P/LP	2	4.21E-04		>47.1	
c.1503C>G	p.Tyr501*	Nonsense	NA	P/LP	1	2.10E-04	4.01E-06	26.2	Yes
c.1575T>G	p.Tyr525*	Nonsense	NA	P/LP	4	8.41E-04		>94.2	
c.1639del	p.547fs	Frameshift	NA	P/LP	4	8.41E-04		>94.2	
c.1641G>A	p.Val547*	Nonsense	NA	P/LP	1	2.10E-04		>23.5	
c.1678del	p.Asp560Thrfs*19	Frameshift	NA	P/LP	1	2.10E-04		>23.5	
c.1687del	p.Val563Cysfs*16	Frameshift	NA	P/LP	1	2.10E-04		>23.5	
c.1729del	p.Trp577Alafs*27	Frameshift	NA	P/LP	1	2.10E-04		>23.5	
c.1755del	p.Asp587Thrfs*4	Frameshift	NA	P/LP	1	2.10E-04		>23.5	
c.1800del	p.Lys600Asnfs*2	Frameshift	NA	P/LP	4	8.41E-04		>94.2	
c.1838dup	p.Asp613Glufs*25	Frameshift	NA	P/LP	2	4.21E-04		>47.1	
c.1892del	p.Phe631fs	Frameshift	NA	P/LP	7	1.47E-03		>164.8	
c.1895del	p.Met632Argfs	Frameshift	NA	P/LP	8	1.68E-03		>188.4	
c.1900del	p.Val634Serfs*29	Frameshift	NA	P/LP	1	2.10E-04		>23.5	
c.1999_2000delinsG	p.Leu667Thrfs*25	Frameshift	NA	P/LP	2	4.21E-04		>47.1	
c.2025_2026del	p.Asp676Profs*16	Frameshift	NA	P/LP	1	2.10E-04		>23.5	
c.2048G>A	p.Trp683*	Nonsense	NA	P/LP	2	4.21E-04		>47.1	
c.2065C>T	p.Gln689*	Nonsense	NA	P/LP	1	2.10E-04		>23.5	
c.2073del	p.Asn691fs	Frameshift	NA	P/LP	1	2.10E-04		>23.5	
c.2096del	p.Pro699Glnfs*55	Frameshift	NA	P/LP	5	1.05E-03		>117.7	
c.2113dup	p.Thr705Asnfs*3	Frameshift	NA	P/LP	3	6.31E-04		>70.6	
c.2132G>A	p.Trp711*	Nonsense	NA	P/LP	1	2.10E-04		>23.5	
c.2149-2del		Frameshift	NA	P/LP	2	4.21E-04		>47.1	
c.2157_2158del	p.Cys719*	Nonsense	NA	P/LP	1	2.10E-04		>23.5	
c.2182G>T	p.Glu728*	Nonsense	NA	P/LP	1	2.10E-04		>23.5	
c.2221del	p.Ala741Glnfs*13	Frameshift	NA	P/LP	1	2.10E-04		>23.5	

c.2240_2245delinsCCTTC	p.Gly747Alafs*7	Frameshift	NA	P/LP	1	2.10E-04		>23.5	
c.2258dup	p.Lys754Glufs*79	Frameshift	NA	P/LP	11	2.31E-03		>259	
c.2259_260insTC	p.Lys754Serfs*69	Frameshift	NA	P/LP	1	2.10E-04		>23.5	
c.2267del	p.Pro756Leufs*66	Frameshift	NA	P/LP	3	6.31E-04		>70.6	
c.2304_2308+2del	p.Ile769Argfs*62	Frameshift	NA	P/LP	2	4.21E-04		>47.1	
c.2353_2357del	p.Glu785Leufs*46	Frameshift	NA	P/LP	1	2.10E-04		>23.5	
c.2371C>T	p.Gln791*	Nonsense	NA	P/LP	3	6.31E-04		>70.6	
c.2373insG	p.Trp792Valfs*41	Frameshift	NA	P/LP	181	3.81E-02		>4262.4	
c.2374insG	p.Trp792fs	Frameshift	NA	P/LP	1	2.10E-04		>23.5	
c.2376G>A	p.Trp792Valfs*41	Frameshift	NA	P/LP	5	1.05E-03		>117.7	
c.2391C>A	p.Tyr797*	Nonsense	NA	P/LP	6	1.26E-03		>141.3	
c.2394_2395insT	p.Gly799Trpfs*34	Frameshift	NA	P/LP	1	2.10E-04		>23.5	
c.2454G>A	p.Trp818*	Nonsense	NA	P/LP	4	8.41E-04		>94.2	
c.2490dup	p.His831Serfs*2	Frameshift	NA	P/LP	1	2.10E-04		>23.5	
c.2524G>A	p.Trp818*	Nonsense	NA	P/LP	1	2.10E-04		>23.5	
c.2524_2525insT		Frameshift	NA	P/LP	1	2.10E-04		>23.5	
c.2526C>G	p.Tyr842*	Nonsense	NA	P/LP	1	2.10E-04		>23.5	
c.2541C>G	p.Tyr847*	Nonsense	NA	P/LP	4	8.41E-04		>94.2	
c.2543_2544del		Frameshift	NA	P/LP	1	2.10E-04		>23.5	
c.2543_2544dup		Frameshift	NA	P/LP	1	2.10E-04		>23.5	
c.2545del		Frameshift	NA	P/LP	1	2.10E-04		>23.5	
c.2550del	p.Asn850Lysfs*29	Frameshift	NA	P/LP	2	4.21E-04		>47.1	
c.2554insT	p.Ile852fs*	Frameshift	NA	P/LP	2	4.21E-04		>47.1	
c.2555_2556insT	p.Gly853fs	Frameshift	NA	P/LP	2	4.21E-04		>47.1	
c.2558del		Frameshift	NA	P/LP	3	6.31E-04		>70.6	
c.2604-2605delTCinsA	p.Ser871Alafs*8	Frameshift	NA	P/LP	1	2.10E-04		>23.5	
c.2670_2671insG	p.Arg891Alafs*160	Frameshift	NA	P/LP	3	6.31E-04		>70.6	
c.2670G>A	p.Trp890*	Nonsense	NA	P/LP	12	2.52E-03		>282.6	
c.2689_2698del	p.Gly897Alafs*24	Frameshift	NA	P/LP	6	1.26E-03		>141.3	
c.2690_2696del		Frameshift	NA	P/LP	1	2.10E-04		>23.5	
c.2710del	p.Tyr904Thrfs*20	Frameshift	NA	P/LP	6	1.26E-03		>141.3	
c.2735del	p.Gly912Alafs*12	Frameshift	NA	P/LP	4	8.41E-04		>94.2	
c.2747G>A	p.Trp916*	Nonsense	NA	P/LP	7	1.47E-03		>164.8	
c.2780_2781del	p.Thr927Ilefs*123	Frameshift	NA	P/LP	1	2.10E-04		>23.5	
c.2792dup	p.Lys932Glufs*119	Frameshift	NA	P/LP	1	2.10E-04		>23.5	
c.2827C>T	p.Arg943*	Nonsense	NA	P/LP	105	2.21E-02	1.21E-05	909.3	Yes
c.2833_2834del	p.Arg945Glyfs*105	Frameshift	NA	P/LP	1	2.10E-04		>23.5	
c.2842_2843del	p.Asn948Tyrfs*102	Frameshift	NA	P/LP	1	2.10E-04		>23.5	
c.2846dup	p.Met949Ilefs*102	Frameshift	NA	P/LP	1	2.10E-04		>23.5	

c.2862_2865del		Frameshift	NA	P/LP	2	4.21E-04		>47.1	
c.2864_2865del	p.Pro955Argfs*95	Frameshift	NA	P/LP	91	1.91E-02		>2143	
c.2869dup	p.Thr957fs*94	Frameshift	NA	P/LP	1	2.10E-04		>23.5	
c.2893C>T	p.Gln965*	Nonsense	NA	P/LP	1	2.10E-04		>23.5	
c.2905C>T	p.Gln969*	Nonsense	NA	P/LP	10	2.10E-03		>235.5	
c.2943_2947del	p.Gln981fs	Frameshift	NA	P/LP	3	6.31E-04		>70.6	
c.2992C>T	p.Gln998*	Nonsense	NA	P/LP	1	2.10E-04		>23.5	
c.3029del	p.Glu1010fs	Frameshift	NA	P/LP	1	2.10E-04		>23.5	
c.3040del	p.Leu1014Trpfs*6	Frameshift	NA	P/LP	1	2.10E-04		>23.5	
c.3100del	p.Ala1034Profs*12	Frameshift	NA	P/LP	1	2.10E-04		>23.5	
c.3124_3125insAA	p.Thr1042Lysfs*5	Frameshift	NA	P/LP	2	4.21E-04		>47.1	
c.3127dup	p.Tyr1043Leufs*8	Frameshift	NA	P/LP	1	2.10E-04		>23.5	
c.3129C>A	p.Tyr1043*	Nonsense	NA	P/LP	1	2.10E-04		>23.5	
c.3163A>T	p.Lys1055*	Nonsense	NA	P/LP	2	4.21E-04		>47.1	
c.3166dup	p.Lys1055*	Nonsense	NA	P/LP	1	2.10E-04		>23.5	
c.3181C>T	p.Gln1061*	Nonsense	NA	P/LP	3	6.31E-04	1.48E-05	21.3	Yes
c.3192dup	p.Lys1065Glufs*12	Frameshift	NA	P/LP	40	8.41E-03		>942	
c.3217dup	p.Arg1073Profs*4	Frameshift	NA	P/LP	1	2.10E-04		>23.5	
c.3226_3227insT	p.Asp1076Valfs*6	Frameshift	NA	P/LP	4	8.41E-04	3.19E-05	13.2	Yes
c.3233G>A	p.Trp1078*	Nonsense	NA	P/LP	12	2.52E-03		>282.6	
c.3257G>A	p.Trp1086*	Nonsense	NA	P/LP	2	4.21E-04		>47.1	
c.3286G>T	p.Glu1096*	Nonsense	NA	P/LP	4	8.41E-04	4.84E-06	86.9	Yes
c.3288G>A	p.Glu1096*	Nonsense	NA	P/LP	3	6.31E-04	4.01E-01	0	No
c.3293G>A	p.Trp1098*	Nonsense	NA	P/LP	2	4.21E-04		>47.1	
c.3297dup	p.Tyr1100Valfs*49	Frameshift	NA	P/LP	3	6.31E-04		>70.6	
c.3300C>A	p.Tyr1100*	Nonsense	NA	P/LP	1	2.10E-04		>23.5	
c.3321dup	p.Lys1108Glufs*41	Frameshift	NA	P/LP	1	2.10E-04		>23.5	
c.3332_3335dup	p.Trp1112Leufs*37	Frameshift	NA	P/LP	1	2.10E-04		>23.5	
c.3334dup	p.Trp1112Leufs*37	Frameshift	NA	P/LP	1	2.10E-04		>23.5	
c.3335G>A	p.Trp1112*	Nonsense	NA	P/LP	1	2.10E-04		>23.5	
c.3372C>A	p.Cys1124*	Nonsense	NA	P/LP	2	4.21E-04	4.13E-06	50.9	Yes
c.3408C>A	p.Tyr1136*	Nonsense	NA	P/LP	2	4.21E-04		>47.1	
c.3414_3415insC	p.Val1139Argfs*10	Frameshift	NA	P/LP	2	4.21E-04		>47.1	
c.3432_3435dup	p.Phe1147Trpfs*3	Frameshift	NA	P/LP	2	4.21E-04		>47.1	
c.3467dup	p.Pro1157Alafs*12	Frameshift	NA	P/LP	1	2.10E-04		>23.5	
c.3476_3477insATTT	p.Phe1159Leufs*11	Frameshift	NA	P/LP	1	2.10E-04		>23.5	
c.3514_3517dup	p.Lys1173Ilefs*2	Frameshift	No	P/LP	2	4.21E-04		>47.1	
c.3617_3618del	p.Gly1206Glufs*35	Frameshift	NA	P/LP	1	2.10E-04		>23.5	
c.3617del	p.Gly1206Valfs*31	Frameshift	NA	P/LP	5	1.05E-03		>117.7	

c.3624_3625insC	p.Lys1209Glnfs*33	Frameshift	NA	P/LP	6	1.26E-03		>141.3	
c.3624del	p.Lys1209Glnfs*28	Frameshift	NA	P/LP	10	2.10E-03		>235.5	
c.3628-41_3628-17del		Frameshift	NA	P/LP	10	2.10E-03		>235.5	
c.3642G>A	p.Trp1214*	Nonsense	NA	P/LP	1	2.10E-04		>23.5	
c.3662_3662del	p.Leu1221Argfs*16	Frameshift	NA	P/LP	7	1.47E-03		>164.8	
c.3690_3691del	p.Phe1230Leufs*11	Frameshift	NA	P/LP	1	2.10E-04		>23.5	
c.3694A>T	p.Lys1232*	Nonsense	NA	P/LP	1	2.10E-04		>23.5	
c.3697C>T	p.Gln1233*	Nonsense	NA	P/LP	25	5.26E-03	8.02E-06	327.5	Yes
c.3702_3703del	p.Leu1236fs	Frameshift	NA	P/LP	1	2.10E-04		>23.5	
c.3712_3713del	p.Leu1238Glyfs*3	Frameshift	NA	P/LP	1	2.10E-04		>23.5	
c.3735del	p.Phe1246fs	Frameshift	NA	P/LP	3	6.31E-04		>70.6	
c.3767_3768del	p.Thr1256Lysfs*9	Frameshift	NA	P/LP	2	4.21E-04		>47.1	
c.3776del	p.Gln1259fs	Frameshift	NA	P/LP	12	2.52E-03		>282.6	
c.3811C>T	p.Arg1271*	Nonsense	NA	P/LP	2	4.21E-04	8.13E-06	25.9	Yes
***MYBPC3* Splice Site Variants in SHaRe**									
c.25+1G>A	NA	Splice	No	P/LP	2	4.21E-04		>47.1	
c.26-2A>G	NA	Splice	No	P/LP	3	6.31E-04	2.74E-05	11.5	Yes
c.407-1G>A	NA	Splice	No	P/LP	1	2.10E-04		>23.5	
c.505+1G>A	NA	Splice	No	P/LP	1	2.10E-04		>23.5	
c.506-1G>A	NA	Splice	No	P/LP	2	4.21E-04		>47.1	
c.506-2A>C	NA	Splice	No	P/LP	4	8.41E-04		>94.2	
c.506-12del	NA	Splice	No	P/LP	4	8.41E-04		>94.2	
c.654+1G>A	NA	Splice	No	P/LP	12	2.52E-03		>282.6	
c.655-1G>A	NA	Splice	No	P/LP	1	2.10E-04		>23.5	
c.655G>C	p.Val219Leu?	Splice	No	P/LP	9	1.89E-03		>211.9	
c.655-?_*26=?Del	NA	Splice	No	P/LP	1	2.10E-04		>23.5	
c.772G>A	p.Glu258Lys	Splice	Yes[5,6]	P/LP	200	4.21E-02	2.20E-05	956.4	Yes
c.772G>C		Splice	No	P/LP	1	2.10E-04		>23.5	
c.821+1G>A	NA	Splice	No	P/LP	13	2.73E-03	2.89E-05	47.3	Yes
c.821+1G>C	NA	Splice	No	P/LP	1	2.10E-04		>23.5	
c.821+2T>C	NA	Splice	No	P/LP	2	4.21E-04		>47.1	
c.906-36G>A	NA	Splice	No	VUS	1	2.10E-04		>23.5	
c.926+8C>T	NA	Splice	No	VUS	2	4.21E-04		>47.1	
c.927-10C>A	NA	Splice	No	VUS	1	2.10E-04		>23.5	
c.927-10C>T	NA	Splice	No	VUS	1	2.10E-04		>23.5	
c.927-1G>C	NA	Splice	No	P/LP	1	2.10E-04		>23.5	
c.927-2A>G	NA	Splice	No	P/LP	176	3.70E-02	8.76E-06	2111.5	Yes
c.927-9G>A	NA	Splice	Yes[3]	P/LP	26	5.47E-03		>612.3	
c.1090G>A	p.Ala364Thr	Splice	Yes[4]	P/LP	6	1.26E-03		>141.3	

c.1090+453C>T	NA	Splice	No	VUS	3	6.31E-04		>70.6	
c.1091-?_2308+?Del	NA	Splice	No	P/LP	2	4.21E-04		>47.1	
c.1091-2A>G	NA	Splice	No	P/LP	1	2.10E-04		>23.5	
c.1224-52G>A	NA	Splice	No	VUS	2	4.21E-04		>47.1	
c.1224-2A>G	NA	Splice	No	P/LP	2	4.21E-04		>47.1	
c.1224-19G>A	NA	Splice	No	VUS	1	2.10E-04		>23.5	
c.1227-13G>A	NA	Splice	No	VUS	2	4.21E-04		>47.1	
c.1227-1G>C	NA	Splice	No	P/LP	1	2.10E-04		>23.5	
c.1227-2A>G	NA	Splice	No	P/LP	4	8.41E-04	3.19E-05	13.2	Yes
c.1351+1G>A	NA	Splice	No	P/LP	2	4.21E-04		>47.1	
c.1351+2T>C	NA	Splice	No	P/LP	1	2.10E-04		>23.5	
c.1457+5G>C	NA	Splice	No	VUS	1	2.10E-04		>23.5	
c.1458-1G>A	NA	Splice	No	P/LP	12	2.52E-03		>282.6	
c.1458-1G>C	NA	Splice	No	P/LP	13	2.73E-03	4.02E-06	340.1	Yes
c.1458-6G>A	NA	Splice	No	VUS	3	6.31E-04		>70.6	
c.1624+1G>A	NA	Splice	No	P/LP	8	1.68E-03		>188.4	
c.1624+206_1624+207ins236	NA	Splice	No	VUS	1	2.10E-04		>23.5	
c.1624+4A>T	NA	Splice	Yes[3]	P/LP	17	3.57E-03		>400.3	
c.1624G>C	p.Glu542Gln (partial)	Splice	Yes[3,4]	P/LP	37	7.78E-03	1.90E-05	204.4	Yes
c.1625-6C>A	NA	Splice	No	VUS	1	2.10E-04		>23.5	
c.1790G>A	p.Arg597Gln?	Splice	No	P/LP	5	1.05E-03	3.00E-05	17.5	Yes
c.1791-1G>A	NA	Splice	No	P/LP	1	2.10E-04		>23.5	
c.1897+5G>A	NA	Splice	No	VUS	2	4.21E-04		>47.1	
c.1898-1G>A	NA	Splice	No	P/LP	1	2.10E-04		>23.5	
c.1928-2A>G	NA	Splice	Yes[3]	P/LP	31	6.52E-03		>730	
c.1928-569G>T	NA	Splice	No	VUS	2	4.21E-04		>47.1	
c.2068-2delA	NA	Splice	No	P/LP	3	6.31E-04		>70.6	
c.2148+158_2737+258del3548insT	NA	Splice	No	VUS	1	2.10E-04		>23.5	
c.2149-1G>A	NA	Splice	No	P/LP	2	4.21E-04		>47.1	
c.2149-2A>C	NA	Splice	No	P/LP	1	2.10E-04		>23.5	
c.2149-9C>A	NA	Splice	No	P/LP	5	1.05E-03		>117.7	
c.2149-5C>T	NA	Splice	No	VUS	2	4.21E-04		>47.1	
c.2149-8C>G	NA	Splice	No	VUS	1	2.10E-04		>23.5	
c.2308+1G>A	NA	Splice	No	P/LP	4	8.41E-04		>94.2	
c.2308+1G>T	NA	Splice	No	P/LP	5	1.05E-03		>117.7	
c.2308+2T>G	NA	Splice	Yes	P/LP	1	2.10E-04		>23.5	
c.2308G>A	NA	Splice	Yes[3]	P/LP	12	2.52E-03	1.61E-05	78.6	Yes
c.2309-2A>G	NA	Splice	Yes	P/LP	13	2.73E-03		>306.1	

c.2413+1del	NA	Splice	No	P/LP	1	2.10E-04		>23.5	
c.2413+1G>A	NA	Splice	No	VUS	1	2.10E-04		>23.5	
c.2414-2A>G	NA	Splice	No	P/LP	1	2.10E-04		>23.5	
c.2603-1G>A	NA	Splice	No	P/LP	1	2.10E-04		>23.5	
c.2737+2_2737+3del	NA	Splice	No	VUS	1	2.10E-04		>23.5	
c.2737+2T>A	NA	Splice	No	P/LP	1	2.10E-04		>23.5	
c.2737+5G>A	NA	Splice	No	VUS	1	2.10E-04		>23.5	
c.2738-2A>T	NA	Splice	No	P/LP	1	2.10E-04		>23.5	
c.2905+1G>A	NA	Splice	Yes[3]	P/LP	12	2.52E-03		>282.6	
c.2905+2T>G	NA	Splice	No	P/LP	1	2.10E-04		>23.5	
c.2905+5G>T	NA	Splice	No	VUS	1	2.10E-04		>23.5	
c.2994+2T>C	NA	Splice	No	P/LP	3	6.31E-04		>70.6	
c.3190+1G>A	NA	Splice	No	P/LP	5	1.05E-03	4.18E-06	125.7	Yes
c.3190+2T>G	NA	Splice	No	P/LP	2	4.21E-04	8.37E-06	25.1	Yes
c.3190+5G>A	NA	Splice	No	P/LP	9	1.89E-03		>211.9	
c.3330+2T>C	NA	Splice	No	P/LP	2	4.21E-04		>47.1	
c.3330+2T>G	NA	Splice	Yes[3]	P/LP	17	3.57E-03	3.19E-05	56	Yes
c.3330+5G>A	NA	Splice	No	VUS	1	2.10E-04		>23.5	
c.3330+5G>C	NA	Splice	Yes	P/LP	8	1.68E-03		>188.4	
c.3331-1G>A	NA	Splice	No	P/LP	3	6.31E-04		>70.6	
c.3331-1G>C	NA	Splice	Yes	P/LP	3	6.31E-04		>70.6	
c.3331-2A>G	NA	Splice	No	P/LP	1	2.10E-04		>23.5	
c.3350+5G>C	NA	Splice	No	VUS	1	2.10E-04		>23.5	
c.3490+1G>A	NA	Splice	No	P/LP	2	4.21E-04		>47.1	
c.3490+1G>T	NA	Splice	No	P/LP	3	6.31E-04		>70.6	
c.3490+5G>A	NA	Splice	No	VUS	1	2.10E-04		>23.5	
c.3490+6G>A	NA	Splice	No	VUS	1	2.10E-04		>23.5	
c.3491-3C>G	NA	Splice	No	VUS	1	2.10E-04		>23.5	
c.3627+1G>A	NA	Splice	No	P/LP	2	4.21E-04		>47.1	
c.3627+2T>G	NA	Splice	No	P/LP	2	4.21E-04		>47.1	
c.3628-1G>A	NA	Splice	No	P/LP	1	2.10E-04		>23.5	
c.3814+1G>A	NA	Splice	No	P/LP	1	2.10E-04		>23.5	
c.3815-1G>A	NA	Splice	No	P/LP	1	2.10E-04	4.12E-06	25.5	Yes

Supplemental Table 1. Truncating *MYBPC3* Mutations in SHaRe (Including Intronic Splice Variants of Unknown Significance)

Sample	Variant	Variant Location	Exon Primers		Intron Primers	
			Result	Sequence	Result	Sequence
HCM 51	c.3331-1 G>C	Intron 30 acceptor	No exon 30 skip	GCAGGTTGTTG ACAAGCCAA...... ACAAGAAGACC ATGGAGTGGTT CACCG	Intron 30 inclusion	CCCTCCCTGCCCCC AGAGTGG....CCAGA CCAGGCATCACCTA TGAGCCACCCAAC
HCM 32, 204	c.3330+2 T>G	Intron 30 donor	Exon 30 skip	GCAGGTTGTTG GAGTGGTTCACC G	No Intron 30 inclusion	
HCM 64	c.3330+5 G>C	Intron 30 donor	Exon 30 skip	GCAGGTTGTTG GAGTGGTTCACC G	No Intron 30 inclusion	
HCM 114	c.2309-2 A>G	Intron 22 acceptor	Exon 22 skip	GCTATCACGCAG CTGCTGTGTGAG A	Intron 22 inclusion	TCTGCACCCCCCCA GCTGCTGTGTGAGA CCGAG...CAGTCAA GGTCATCGACGTGC CAGACGCACCT
HCM 205	c.2308+2 T>G	Intron 22 donor	Exon 22 skip	GCTATCACGCAG CTGCTGTGTGAG A	Intron 22 inclusion	TCTGCACCCCCCCA GCTGCTGTGTGAGA CCGAG...ACAGTCA AGGTCATCGACGTG CCAGACGCACCT
HCM 213	c.1224-19 G>A	Intron 12 acceptor	No exon 22 skip	GGACCCCGAGG GACTC...AAGAG CACAGCCTTTCA GAAGA	No intron 12 inclusion	

Supplemental Table 2. Summary of sequencing results for selected splice variants using exon and intron primers.

MYBPC3 Non-Synonmyous Missense and Non-Truncating Variants in SHaRe									
**Nucleotide Variant**	**Amino Acid Variant**	**Potential Exonic Splice Site**	**MyBP-C Domain**	**Category**	**SHaRe Number of Cases**	**SHaRe Case Frequency**	**gnomAD Allele Frequency**	**Odds Ratio (SHaRe Enrichment)**	**Ito, et al.[7], Positive Mini-Gene Assay**
c.13G>C	p.Gly5Arg	No	0	B/LB	2	4.20E-04	3.19E-04	0.7	
c.13G>T	p.Gly5Trp	No	0	VUS	1	2.10E-04	4.31E-06	24.4	
c.50G>A	p.Arg17Gln	No	0	B/LB	1	2.10E-04	8.48E-05	1.2	
c.94G>A	p.Glu32Lys	No	0	B/LB	1	2.10E-04	1.58E-04	0.7	
c.103C>T	p.Arg35Trp	No	0	VUS	2	4.20E-04	2.49E-05	8.4	
c.104G>A	p.Arg35Gln	No	0	VUS	3	6.30E-04	6.64E-05	4.8	
c.133G>A	p.Gly45Arg	No	0	VUS	1	2.10E-04	2.08E-05	5.1	
c.175A>G	p.Thr59Ala	No	0	VUS	1	2.10E-04		>23.5	
c.184A>C	p.Thr62Pro	No	0	B/LB	1	2.10E-04	1.50E-04	0.7	
c.188G>A	p.Arg63Gln	No	0	VUS	1	2.10E-04	2.66E-05	3.9	
c.223G>A	p.Asp75Asn	No	0	VUS	5	1.05E-03	1.75E-05	30	
c.327_332del	p.Del110_111	No	0	VUS	1	2.10E-04		>23.5	
c.340A>G	p.Thr114Ala	No	0	VUS	1	2.10E-04		>23.5	
c.362C>T	p.Pro121Leu	No	0	B/LB	1	2.10E-04	6.15E-05	1.7	
c.373G>T	p.Ala125Ser	No	0	VUS	1	2.10E-04	3.19E-05	3.3	
c.442G>A	p.Gly148Arg	Yes	0	VUS	18	3.78E-03	6.48E-05	29.2	Yes
c.461T>C	p.Ile154Thr	No	0	B/LB	1	2.10E-04	9.68E-05	1.1	
c.464G>T	p.Gly155Val	No	1	VUS	1	2.10E-04		>23.5	
c.472G>A	p.Val158Met	No	1	B/LB	3	6.30E-04	6.62E-02	0	
c.478C>T	p.Arg160Trp	Yes	1	B/LB	1	2.10E-04	1.45E-03	0.1	No
c.481C>T	p.Pro161Ser	No	1	P/LP	10	2.10E-03	5.81E-06	180.9	
NA	p.Glu165Gln	No	1	VUS	1	2.10E-04		>23.5	
c.495G>C	p.Glu165Asp	No	1	B/LB	1	2.10E-04	8.94E-05	1.2	
c.497T>G	p.Val166Gly	No	1	VUS	1	2.10E-04		>23.5	
c.518C>A	p.Thr173Asn	No	1	VUS	1	2.10E-04		>23.5	
c.529C>T	p.Arg177Cys	No	1	VUS	2	4.20E-04	6.41E-05	3.3	
c.530G>A	p.Arg177His	No	1	B/LB	3	6.30E-04	1.20E-03	0.3	
c.532G>A	p.Val178Met	No	1	VUS	1	2.10E-04		>23.5	
c.557C>T	p.Pro186Leu	No	1	B/LB	2	4.20E-04	4.51E-05	4.7	
c.565G>A	p.Val189Ile	No	1	B/LB	4	8.39E-04	2.46E-03	0.2	
c.624G>C	p.Gln208His	No	1	B/LB	1	2.10E-04	2.18E-04	0.5	
c.636C>G	p.Ser212Arg	Yes	1	VUS	2	4.20E-04		>47.1	Yes
c.640G>A	p.Asp214Asn	No	1	VUS	2	4.20E-04	3.30E-05	6.4	
c.646G>A	p.Ala216Thr	No	1	B/LB	3	6.30E-04	4.01E-04	0.8	

c.649A>C	p.Ser217Arg	No	1	VUS	1	2.10E-04		>23.5	
c.649A>G	p.Ser217Gly	No	1	B/LB	9	1.89E-03	1.69E-03	0.6	
c.659A>G	p.Tyr220Cys	No	1	VUS	1	2.10E-04	2.18E-05	4.8	
c.706A>G	p.Ser236Gly	No	1	B/LB	5	1.05E-03	9.64E-02	0	
c.710A>C	p.Tyr237Ser	Yes	1	P/LP	2	4.20E-04		>47.1	No
c.756C>A	p.Phe252Leu	No	1	VUS	1	2.10E-04		>23.5	
c.767T>G	p.Val256Gly	No	M	VUS	1	2.10E-04		>23.5	
c.787G>A	p.Gly263Arg	No	M	B/LB	1	2.10E-04	8.38E-05	1.3	
c.818G>A	p.Arg273His	No	M	VUS	3	6.30E-04	9.18E-05	3.4	
c.833G>A	p.Gly278Glu	No	M	B/LB	1	2.10E-04	1.33E-03	0.1	
c.884T>C	p.Phe295Ser	No	M	VUS	1	2.10E-04		>23.5	
c.931T>A	p.Ser311Thr	No	M	VUS	1	2.10E-04		>23.5	
c.931T>C	p.Ser311Pro	No	M	VUS	1	2.10E-04		>23.5	
c.961G>A	p.Val321Met	No	M	B/LB	4	6.30E-04	3.30E-04	1.3	
c.964T>C	p.Trp322Arg	No	M	VUS	1	2.10E-04		>23.5	
c.977G>A	p.Arg326Gln	No	M	B/LB	4	8.39E-04	4.36E-03	0.1	
c.1000G>A	p.Glu334Lys	Yes	M	B/LB	2	4.20E-04	2.37E-04	0.9	No
c.1021G>A	p.Gly341Ser	No	M	VUS	1	2.10E-04	5.00E-05	2.1	
c.1069C>T	p.Arg357Cys	No	M	VUS	1	2.10E-04	8.03E-06	13.1	
c.1084A>C	p.Ser362Arg	No	2	VUS	1	2.10E-04		>23.5	
c.1112C>G	p.Pro371Arg	No	2	P/LP	11	2.31E-03		>259	
c.1147C>G	p.Leu383Val	No	2	B/LB	1	2.10E-04	7.71E-05	1.4	
c.1153G>A	p.Val385Met	No	2	VUS	1	2.10E-04	1.22E-05	8.6	
c.1217G>A	p.Ser406Asn	No	2	VUS	1	2.10E-04	4.67E-06	22.5	
c.1218C>A	p.Ser406Arg	No	2	VUS	1	2.10E-04		>23.5	
c.1238A>G	p.Glu413Gly	No	2	VUS	1	2.10E-04		>23.5	
c.1246G>A	p.Gly416Ser	No	2	B/LB	1	2.10E-04	1.06E-04	1	
c.1286C>T	p.Ala429Val	No	2	B/LB	1	2.10E-04	1.67E-04	0.6	
c.1294G>A	p.Ala432Thr	No	2	VUS	1	2.10E-04	2.46E-05	4.3	
c.1321G>A	p.Glu441Lys	No	2	B/LB	7	1.47E-03	1.47E-04	5	
c.1408C>T	p.Arg470Trp	No	3	VUS	3	6.30E-04		>70.6	
c.1418T>C	p.Phe473Ser	No	3	VUS	1	2.10E-04		>23.5	
c.1456T>G	p.Trp486Gly	Yes	3	VUS	3	6.31E-04		>70.6	No
c.1468G>A	p.Gly490Arg	No	3	B/LB	7	1.47E-03	2.14E-04	3.4	
c.1471G>A	p.Val491Met	No	3	VUS	3	6.30E-04	5.62E-05	5.6	
c.1483C>G	p.Arg495Gly	No	3	P/LP	13	2.73E-03	4.01E-06	340.6	
c.1483C>T	p.Arg495Trp	No	3	P/LP	3	6.30E-04		>70.6	
c.1484G>A	p.Arg495Gln	No	3	P/LP	33	6.93E-03	2.41E-05	144.1	
c.1501T>C	p.Tyr501His	No	3	VUS	1	2.10E-04		>23.5	
c.1504C>T	p.Arg502Trp	No	3	P/LP	67	1.41E-02	4.63E-05	152.1	

c.1505G>A	p.Arg502Gln	No	3	P/LP	46	9.65E-03		>1083.3	
c.1509C>G	p.Phe503Leu	No	3	P/LP	2	4.20E-04		>47.1	
c.1513_1515del	p.Lys505del	No	3	VUS	1	2.10E-04		>23.5	
c.1519G>A	p.Gly507Arg	No	3	B/LB	6	1.26E-03	6.56E-04	1	
c.1544A>G	p.Asn515Ser	No	3	B/LB	1	2.10E-04	1.25E-04	0.8	
c.1564G>A	p.Ala522Thr	No	3	B/LB	1	2.10E-04	4.88E-04	0.2	
c.1568G>T	p.Gly523Val	No	3	VUS	3	6.30E-04		>70.6	
c.1573T>C	p.Tyr525His	No	3	VUS	1	2.10E-04		>23.5	
c.1574A>C	p.Tyr525Ser	No	3	VUS	1	2.10E-04		>23.5	
c.1580T>C	p.Leu527Pro	No	3	VUS	2	4.20E-04	8.05E-06	26.1	
c.1591G>C	p.Gly531Arg	No	3	P/LP	13	2.73E-03	1.80E-05	76.1	
c.1601C>A	p.Ala534Glu	No	3	VUS	1	2.10E-04		>23.5	
c.1607C>G	p.Ala536Gly	No	3	VUS	1	2.10E-04		>23.5	
c.1615A>G	p.Ile539Val	No	3	VUS	1	2.10E-04	3.36E-05	3.1	
c.1684G>A	p.Ala562Thr	No	4	VUS	1	2.10E-04		>23.5	
c.1685C>T	p.Ala562Val	No	4	VUS	1	2.10E-04	4.02E-06	26.2	
c.1696T>C	p.Cys566Arg	Yes	4	VUS	3	6.30E-04		>70.6	No
c.1720C>T	p.Arg574Trp	No	4	VUS	1	2.10E-04	5.27E-05	2	
c.1721G>A	p.Arg574Gln	Yes	4	VUS	1	2.10E-04	6.48E-05	1.6	No
c.1765C>G	p.Arg589Gly	No	4	P/LP	7	1.47E-03		>164.8	
c.1765C>T	p.Arg589Cys	No	4	VUS	2	4.20E-04	9.97E-06	21.1	
c.1766G>A	p.Arg589His	No	4	VUS	1	2.10E-04	1.02E-05	10.3	
c.1783A>G	p.Ile595Val	No	4	VUS	2	4.20E-04		>47.1	
c.1786G>A	p.Gly596Arg	No	4	B/LB	2	4.20E-04	1.03E-04	2	
c.1789C>T	p.Arg597Trp	Yes	4	VUS	4	8.41E-04	2.97E-05	14.2	No
c.1808T>C	p.Ile603Thr	No	4	VUS	1	2.10E-04		>23.5	
c.1809T>G	p.Ile603Met	No	4	VUS	2	4.20E-04	1.42E-05	14.8	
c.1813G>A	p.Asp605Asn	No	4	B/LB	3	6.30E-04	1.89E-04	1.7	
c.1814A>G	p.Asp605Gly	No	4	VUS	1	2.10E-04	5.64E-05	1.9	
c.1822C>T	p.Pro608Ser	No	4	VUS	1	2.10E-04	9.18E-05	1.1	
c.1828G>A	p.Asp610Asn	No	4	VUS	1	2.10E-04	1.35E-05	7.8	
c.1828G>C	p.Asp610His	No	4	VUS	5	1.05E-03	3.15E-05	16.7	
c.1831G>A	p.Glu611Lys	No	4	VUS	4	8.39E-04	2.35E-05	17.9	
c.1855G>A	p.Glu619Lys	No	4	B/LB	12	2.52E-03	4.64E-04	2.7	
c.1871A>G	p.Asn624Ser	No	4	VUS	1	2.10E-04	2.20E-05	4.8	
c.1955C>G	p.Pro652Arg	No	5	VUS	1	2.10E-04	4.01E-06	26.2	
c.2003G>A	p.Arg668His	No	5	B/LB	2	4.20E-04	1.21E-04	1.7	
c.2011G>A	p.Val671Ile	No	5	VUS	1	2.10E-04	4.02E-06	26.2	
c.2030C>T	p.Pro677Leu	No	5	VUS	3	6.30E-04		>70.6	

c.2056G>C	p.Ala686Pro	No	5	VUS	1	2.10E-04	7.55E-06	13.9	
c.2077G>T	p.Ala693Ser	No	5	VUS	1	2.10E-04	5.62E-05	1.9	
c.2078C>T	p.Ala693Val	No	5	VUS	1	2.10E-04	4.01E-06	26.2	
c.2179C>T	p.Val727Met	No	5	VUS	2	4.20E-04		>47.1	
c.2197C>T	p.Arg733Cys	No	5	B/LB	1	2.10E-04	6.07E-05	1.7	
c.2198G>A	p.Arg733His	No	5	VUS	1	2.10E-04	5.00E-05	2.1	
c.2210C>T	p.Thr737Met	No	5	VUS	1	2.10E-04	7.49E-05	1.4	
c.2221G>T	p.Ala741Ser	No	5	VUS	1	2.10E-04	4.01E-06	26.2	
c.2234A>G	p.Asp745Gly	Yes	5	VUS	2	4.20E-04		>47.1	No
c.2252T>A	p.Val751Asp	No	5	VUS	1	2.10E-04		>23.5	
c.2311G>A	p.Val771Met	Yes	5	VUS	2	4.20E-04	3.96E-05	5.3	No
c.2312T>C	p.Val771Ala	No	5	VUS	1	2.10E-04		>23.5	
c.2324C>G	p.Pro775Arg	No	6	VUS	1	2.10E-04		>23.5	
c.2356G>T	p.Asp786Tyr	No	6	VUS	1	2.10E-04		>23.5	
c.2374T>C	p.Trp792Arg	No	6	P/LP	16	3.36E-03		>376.8	
c.2381C>T	p.Pro794Leu	No	6	B/LB	1	2.10E-04	1.36E-04	0.8	
c.2398G>A	p.Gly800Arg	No	6	VUS	1	2.10E-04	3.14E-05	3.3	
c.2429G>A	p.Arg810His	No	6	P/LP	18	3.78E-03	4.82E-05	39.3	
c.2429G>T	p.Arg810Leu	No	6	P/LP	6	1.26E-03		>141.3	
c.2431_2433del	p.Lys811del	No	6	VUS	1	2.10E-04		>23.5	
c.2432A>G	p.Lys811Arg	No	6	VUS	2	2.10E-04		>47.1	
c.2435A>G	p.Lys812Arg	No	6	VUS	1	2.10E-04		>23.5	
c.2440_2442del	p.Lys814del	No	6	P/LP	25	1.26E-03		>588.7	
c.2449C>G	p.Arg817Gly	No	6	VUS	3	6.30E-04		>70.6	
c.2449C>T	p.Arg817Trp	No	6	VUS	1	2.10E-04	4.01E-06	26.2	
c.2450G>A	p.Arg817Gln	No	6	VUS	1	2.10E-04	1.78E-05	5.9	
c.2456T>C	p.Met819Thr	No	6	VUS	1	2.10E-04	4.01E-06	26.2	
c.2458C>T	p.Arg820Trp	No	6	VUS	1	2.10E-04	4.01E-06	26.2	
c.2459G>A	p.Arg820Gln	No	6	P/LP	4	8.39E-04	2.01E-05	21	
c.2497G>A	p.Ala833Thr	No	6	B/LB	3	6.30E-04	1.45E-03	0.2	
c.2533C>A	p.Arg845Ser	No	6	VUS	3	6.30E-04		>70.6	
c.2543C>A	p.Ala848Glu	No	6	P/LP	4	8.39E-04		>94.2	
c.2543C>G	p.Ala848Gly	No	6	VUS	1	2.10E-04	8.08E-06	13	
c.2550C>G	p.Asn850Lys	No	6	VUS	1	2.10E-04		>23.5	
c.2552C>T	p.Ala851Val	No	6	B/LB	1	2.10E-04	6.12E-05	1.7	
c.2558G>T	p.Gly853Val	No	6	VUS	1	2.10E-04		>23.5	
c.2573G>A	p.Ser858Asn	No	6	P/LP	7	1.47E-03	7.31E-06	100.7	
c.2614G>A	p.Glu872Lys	No	6	B/LB	2	4.20E-04	4.78E-04	0.4	
c.2618C>A	p.Pro873His	No	7	B/LB	3	6.30E-04	7.22E-05	4.4	
c.2672G>A	p.Arg891Gln	No	7	VUS	1	2.10E-04	4.09E-05	2.6	

c.2686G>A	p.Val896Met	No	7	B/LB	8	1.68E-03	6.19E-03	0.1	
c.2715C>A	p.Ser905Arg	No	7	VUS	1	2.10E-04		>23.5	
c.2728C>A	p.Pro910Thr	No	7	B/LB	2	4.20E-04	1.45E-04	1.5	
c.2761C>G	p.Gln921Glu	No	7	B/LB	1	2.10E-04	1.50E-04	0.7	
c.2765G>A	p.Gly922Glu	No	7	VUS	1	2.10E-04	2.19E-05	4.8	
c.2783C>T	p.Ser928Leu	No	7	VUS	1	2.10E-04	2.42E-05	4.3	
c.2834G>A	p.Arg945Leu	No	7	VUS	1	2.10E-04	1.22E-05	8.6	
c.2839C>A	p.His947Asn	No	7	VUS	1	2.10E-04		>23.5	
c.2849C>T	p.Ala950Val	No	7	VUS	1	2.10E-04	4.10E-06	25.6	
c.2860G>A	p.Ala954Thr	No	7	VUS	1	2.10E-04	1.28E-05	8.2	
c.2870C>G	p.Thr957Ser	No	7	B/LB	4	8.39E-04	9.48E-04	0.4	
c.2873C>T	p.Thr958Ile	No	7	B/LB	3	4.20E-04	1.62E-04	1.9	
c.2882C>T	p.Pro961Leu	No	7	B/LB	1	2.10E-04	5.05E-05	2.1	
c.2908C>T	p.Arg970Trp	No	7	VUS	1	2.10E-04		>23.5	
c.2909G>A	p.Arg970Gln	No	7	VUS	1	2.10E-04	5.09E-05	2.1	
c.2914C>T	p.Arg972Trp	No	8	B/LB	1	2.10E-04	2.95E-04	0.4	
c.2915G>A	p.Arg972Gln	No	8	VUS	1	2.10E-04	4.85E-05	2.2	
c.2927C>T	p.Pro976Leu	No	8	VUS	1	2.10E-04		>23.5	
c.2980C>T	p.Leu994Phe	No	8	VUS	2	4.20E-04	8.46E-05	2.5	
c.2992C>G	p.Gln998Glu	No	8	B/LB	5	1.05E-03	6.36E-03	0.1	
c.3004C>T	p.Arg1002Trp	No	8	B/LB	3	6.30E-04	7.79E-04	0.4	
c.3005G>A	p.Arg1002Gln	No	8	VUS	2	4.20E-04	5.99E-05	3.5	
c.3019T>C	p.Trp1007Arg	No	8	VUS	1	2.10E-04		>23.5	
c.3064C>T	p.Arg1022Cys	No	8	VUS	3	6.30E-04	1.08E-05	29.3	
c.3065G>A	p.Arg1022His	No	8	VUS	2	4.20E-04	7.19E-06	29.2	
c.3065G>C	p.Arg1022Pro	No	8	VUS	6	1.26E-03	2.52E-05	25.1	
c.3098G>A	p.Arg1033Gln	Yes	8	VUS	1	2.10E-04	8.12E-06	12.9	No
c.3106C>T	p.Arg1036Cys	Yes	8	B/LB	1	2.10E-04	5.53E-04	0.2	No
c.3137C>T	p.Thr1046Met	No	8	VUS	1	2.10E-04	6.60E-05	1.6	
c.3188_3190del	p.Val1063del	No	8	VUS	1	2.10E-04		>23.5	
c.3218G>C	p.Arg1073Pro	No	9	VUS	1	2.10E-04		>23.5	
c.3277G>T	p.Gly1093Cys	No	9	VUS	2	2.10E-04	3.85E-05	5.5	
c.3284C>T	p.Thr1095Met	No	9	VUS	1	2.10E-04	2.42E-05	4.3	
c.3326C>T	p.Thr1109Ile	No	9	B/LB	2	4.20E-04	1.18E-04	1.8	
c.3335_3337dup	p.Trp1112Dup	No	9	VUS	1	2.10E-04		>23.5	
c.3340_3342del	p.Thr1114del	No	9	VUS	1	2.10E-04		>23.5	
c.3343G>A	p.Val1115Ile	No	9	VUS	1	2.10E-04	1.25E-05	8.4	
c.3370T>C	p.Cys1124Arg	No	9	VUS	3	6.30E-04	3.19E-05	9.9	
c.3373G>A	p.Val1125Met	No	9	VUS	1	2.10E-04	2.56E-05	4.1	

c.3392T>C	p.Ile1131Thr	No	9	B/LB	5	1.05E-03	8.40E-04	0.6	
c.3407_3409del	p.Tyr1136del	No	9	VUS	3	6.30E-04		>70.6	
c.3413G>A	p.Arg1138His	No	9	B/LB	2	4.20E-04	1.14E-03	0.2	
c.3413G>C	p.Arg1138Pro	Yes	9	VUS	2	4.20E-04		>47.1	No
c.3452C>T	p.Ala1151Val	No	9	VUS	4	8.39E-04	3.82E-05	11	
c.3480C>G	p.Ile1160Met	No	9	VUS	1	2.10E-04		>23.5	
c.3535G>A	p.Glu1179Lys	No	9	B/LB	3	6.30E-04	4.12E-04	0.8	
c.3551C>A	p.Thr1184Asn	No	10	VUS	2	4.20E-04		>47.1	
c.3560T>G	p.Leu1187Arg	No	10	VUS	2	4.20E-04		>47.1	
c.3581C>T	p.Ala1194Val	No	10	B/LB	1	2.10E-04	1.04E-04	1	
c.3584G>T	p.Gly1195Val	No	10	VUS	1	2.10E-04		>23.5	
c.3599T>C	p.Leu1200Pro	No	10	VUS	2	4.20E-04		>47.1	
c.3605G>A	p.Cys1202Tyr	No	10	VUS	1	2.10E-04		>23.5	
c.3613C>T	p.Arg1205Trp	No	10	VUS	1	2.10E-04	4.03E-06	26.1	
c.3614G>A	p.Arg1205Gln	No	10	VUS	1	2.10E-04	2.15E-05	4.9	
c.3640T>C	p.Trp1214Arg	No	10	VUS	2	2.10E-04		>47.1	
c.3642G>T	p.Trp1214Cys	No	10	VUS	1	2.10E-04		>23.5	
c.3682C>T	p.Arg1228Cys	Yes	10	B/LB	1	2.10E-04	2.14E-04	0.5	No
c.3713T>C	p.Leu1238Pro	No	10	P/LP	1	2.10E-04		>23.5	
c.3728C>G	p.Pro1243Arg	No	10	VUS	1	2.10E-04		>23.5	
c.3737T>C	p.Phe1246Ser	No	10	VUS	1	2.10E-04		>23.5	
c.3742_3759dup	p.Gly1248_Cys1253dup	No	10	P/LP	9	1.47E-03		>211.9	
c.3742G>A	p.Gly1248Arg	Yes	10	VUS	1	2.10E-04	3.21E-05	3.3	No
c.3751T>C	p.Tyr1251His	No	10	VUS	3	6.30E-04		>70.6	
c.3763G>A	p.Ala1255Thr	Yes	10	VUS	3	6.30E-04	6.78E-05	4.7	No
c.3763G>C	p.Ala1255Pro	No	10	VUS	1	2.10E-04		>23.5	
c.3767_3769del	p.Thr1256del	No	10	P/LP	5	1.05E-03		>117.7	
c.3771C>A	p.Asn1257Lys	No	10	P/LP	3	6.30E-04		>70.6	
c.3787C>T	p.Arg1263Trp	No	10	B/LB	1	2.10E-04	9.65E-05	1.1	
c.3791G>T	p.Cys1264Phe	No	10	VUS	1	2.10E-04		>23.5	
c.3797G>A	p.Cys1266Tyr	No	10	VUS	1	2.10E-04		>23.5	
c.3808G>A	p.Val1270Met	No	10	VUS	1	2.10E-04		>23.5	
c.3812G>A	p.Arg1271Gln	No	10	VUS	1	2.10E-04	8.13E-06	12.9	
**Synonymous *MYBPC3* Variants in SHaRe**									
c.12G>A	p.Pro4=	No	0	B/LB	1	2.10E-04	1.03E-04	1	
c.405A>G	p.Lys135=	Yes	1	VUS	1	2.10E-04		>23.5	No
c.471C>T	p.Phe157=	Yes	1	B/LB	1	2.10E-04	4.36E-04	0.2	Yes
c.492C>T	p.Gly164=	No	1	B/LB	3	6.30E-04	2.07E-02	0	
c.786C>T	p.Thr262=	No	2	B/LB	1	2.10E-04	9.00E-02	0	

c.933G>C	p.Ser311=	No	2	B/LB	1	2.10E-04	1.33E-04	0.8	
c.1290C>T	p.Asp430=	No	2	B/LB	1	2.10E-04	2.54E-05	4.1	
c.1566G>A	p.Ala522=	No	3	B/LB	2	4.20E-04	2.14E-04	1	
c.1608T>A	p.Ala536=	No	3	B/LB	2	4.20E-04	1.33E-03	0.2	
c.1830C>T	p.Asp610=	No	4	B/LB	1	2.10E-04	2.70E-05	3.9	
c.2178C>T	p.Arg726=	No	5	B/LB	1	2.10E-04	2.42E-05	4.3	
c.2274C>T	p.Gly758=	Yes	5	VUS	4	8.39E-04	4.01E-06	104.8	No
c.2319C>T	p.Asp773=	No	5	B/LB	1	2.10E-04	1.32E-05	8	
c.2997C>T	p.Gly999=	Yes	8	VUS	1	2.10E-04		>23.5	No
c.3087C>T	p.Ile1029=	No	8	B/LB	1	2.10E-04		>23.5	
c.3315C>A	p.Ala1105=	No	9	B/LB	1	2.10E-04	1.71E-04	0.6	
c.3753T>C	p.Tyr1251=	No	10	B/LB	1	2.10E-04	1.04E-04	1	

Supplemental Table 3. Non-Truncating *MYBPC3* Variants in SHaRe

GnomAD *MYBPC3* Variants Exceeding Population Frequency of 4 x 10^{-5}				
**Nucleotide Variant**	**Amino Acid Variant**	**Allele Count**	**Allele Number**	**Allele Frequency**
c.3787C>T	p.Arg1263Trp	27	279650	9.65E-05
c.3763G>A	p.Ala1255Thr	19	280328	6.78E-05
c.3682C>T	p.Arg1228Cys	60	280636	2.14E-04
c.3676C>T	p.Arg1226Cys	12	249220	4.82E-05
c.3581C>T	p.Ala1194Val	26	249002	1.04E-04
c.3569G>A	p.Arg1190His	13	280282	4.64E-05
c.3535G>A	p.Glu1179Lys	115	278922	4.12E-04
c.3472G>A	p.Val1158Ile	31	236522	1.31E-04
c.3415G>A	p.Val1139Ile	22	268026	8.21E-05
c.3413G>A	p.Arg1138His	306	268590	1.14E-03
c.3412C>T	p.Arg1138Cys	11	268516	4.10E-05
c.3392T>C	p.Ile1131Thr	229	272528	8.40E-04
c.3384G>C	p.Glu1128Asp	23	273044	8.42E-05
c.3362G>A	p.Arg1121His	21	272608	7.70E-05
c.3331G>A	p.Glu1111Lys	2	31344	6.38E-05
c.3326C>T	p.Thr1109Ile	27	228010	1.18E-04
c.3323A>C	p.Lys1108Thr	16	259538	6.16E-05
c.3274G>A	p.Val1092Ile	9	202588	4.44E-05
c.3232T>C	p.Trp1078Arg	12	241568	4.97E-05
c.3154A>G	p.Met1052Val	11	272206	4.04E-05
c.3148G>A	p.Glu1050Lys	22	272720	8.07E-05
c.3137C>T	p.Thr1046Met	16	242472	6.60E-05
c.3107G>A	p.Arg1036His	17	276658	6.14E-05
c.3106C>T	p.Arg1036Cys	153	276826	5.53E-04
c.3049G>A	p.Glu1017Lys	22	277518	7.93E-05
c.3005G>A	p.Arg1002Gln	16	267108	5.99E-05
c.3004C>T	p.Arg1002Trp	208	266896	7.79E-04
c.2992C>G	p.Gln998Glu	1371	215704	6.36E-03
c.2980C>T	p.Leu994Phe	19	224572	8.46E-05
c.2938C>T	p.Arg980Cys	2	31394	6.37E-05
c.2915G>A	p.Arg972Gln	9	185428	4.85E-05
c.2914C>T	p.Arg972Trp	64	217182	2.95E-04
c.2909G>A	p.Arg970Gln	11	216302	5.09E-05
c.2882C>T	p.Pro961Leu	13	257588	5.05E-05
c.2873C>T	p.Thr958Ile	43	265618	1.62E-04
c.2870C>G	p.Thr957Ser	253	266912	9.48E-04
c.2801T>C	p.Leu934Pro	2	31376	6.37E-05
c.2771C>T	p.Thr924Ile	13	276810	4.70E-05
c.2762A>T	p.Gln921Leu	10	242022	4.13E-05

c.2761C>G	p.Gln921Glu	41	273870	1.50E-04
c.2728C>A	p.Pro910Thr	25	172572	1.45E-04
c.2686G>A	p.Val896Met	1270	205022	6.19E-03
c.2684G>A	p.Arg895His	16	208502	7.67E-05
c.2672G>A	p.Arg891Gln	9	220182	4.09E-05
c.2654C>T	p.Thr885Met	3	31376	9.56E-05
c.2618C>T	p.Pro873Leu	13	207756	6.26E-05
c.2618C>A	p.Pro873His	15	207756	7.22E-05
c.2614G>A	p.Glu872Lys	98	205060	4.78E-04
c.2602G>A	p.Gly868Ser	14	227862	6.14E-05
c.2560A>G	p.Met854Val	3	31382	9.56E-05
c.2552C>T	p.Ala851Val	17	277812	6.12E-05
c.2504G>T	p.Arg835Leu	14	280406	4.99E-05
c.2498C>T	p.Ala833Val	712	280508	2.54E-03
c.2497G>A	p.Ala833Thr	408	280532	1.45E-03
c.2381C>T	p.Pro794Leu	28	205190	1.36E-04
c.2269G>A	p.Val757Met	16	249212	6.42E-05
c.2242G>A	p.Val748Ile	10	249192	4.01E-05
c.2210C>T	p.Thr737Met	21	280356	7.49E-05
c.2200A>G	p.Ser734Gly	10	248872	4.02E-05
c.2198G>A	p.Arg733His	14	280128	5.00E-05
c.2197C>T	p.Arg733Cys	17	280042	6.07E-05
c.2179G>A	p.Val727Met	19	279250	6.80E-05
c.2176C>T	p.Arg726Cys	16	278974	5.74E-05
c.2125G>A	p.Asp709Asn	2	31392	6.37E-05
c.2077G>T	p.Ala693Ser	14	249134	5.62E-05
c.2003G>A	p.Arg668His	34	280486	1.21E-04
c.1999C>G	p.Leu667Val	10	249122	4.01E-05
c.1964T>C	p.Ile655Thr	2	31392	6.37E-05
c.1915G>A	p.Val639Ile	13	224214	5.80E-05
c.1855G>A	p.Glu619Lys	121	260592	4.64E-04
c.1822C>T	p.Pro608Ser	20	217952	9.18E-05
c.1814A>G	p.Asp605Gly	12	212592	5.64E-05
c.1813G>A	p.Asp605Asn	46	243934	1.89E-04
c.1786G>A	p.Gly596Arg	21	203560	1.03E-04
c.1721G>A	p.Arg574Gln	18	277928	6.48E-05
c.1720C>T	p.Arg574Trp	13	246750	5.27E-05
c.1564G>A	p.Ala522Thr	137	280474	4.88E-04
c.1544A>G	p.Asn515Ser	35	280644	1.25E-04
c.1519G>A	p.Gly507Arg	184	280644	6.56E-04
c.1471G>A	p.Val491Met	14	249120	5.62E-05

c.1468G>A	p.Gly490Arg	60	280454	2.14E-04
c.1373G>A	p.Arg458His	13	276446	4.70E-05
c.1370C>T	p.Thr457Met	49	245016	2.00E-04
c.1321G>A	p.Glu441Lys	41	278096	1.47E-04
c.1286C>T	p.Ala429Val	46	276102	1.67E-04
c.1246G>A	p.Gly416Ser	29	274848	1.06E-04
c.1243A>C	p.Ile415Leu	2	31388	6.37E-05
c.1147C>G	p.Leu383Val	19	246460	7.71E-05
c.1144C>T	p.Arg382Trp	1178	277868	4.24E-03
c.1091C>T	p.Ala364Val	10	241648	4.14E-05
c.1021G>A	p.Gly341Ser	14	280044	5.00E-05
c.1000G>A	p.Glu334Lys	66	278690	2.37E-04
c.977G>A	p.Arg326Gln	1200	275286	4.36E-03
c.961G>A	p.Val321Met	89	269910	3.30E-04
c.842G>A	p.Arg281Gln	11	217880	5.05E-05
c.833G>A	p.Gly278Glu	290	217654	1.33E-03
c.818G>A	p.Arg273His	16	174260	9.18E-05
c.814C>T	p.Arg272Cys	9	208346	4.32E-05
c.787G>A	p.Gly263Arg	18	214866	8.38E-05
c.758A>G	p.Asn253Ser	4	31362	1.28E-04
c.713G>A	p.Arg238His	25	247534	1.01E-04
c.706A>G	p.Ser236Gly	26894	278870	9.64E-02
c.684T>G	p.Asp228Glu	22	278754	7.89E-05
c.682G>A	p.Asp228Asn	22	278398	7.90E-05
c.667G>A	p.Glu223Lys	23	245622	9.36E-05
c.649A>G	p.Ser217Gly	460	271656	1.69E-03
c.646G>A	p.Ala216Thr	109	271662	4.01E-04
c.643C>T	p.Arg215Cys	28	240996	1.16E-04
c.624G>C	p.Gln208His	60	275114	2.18E-04
c.565G>A	p.Val189Ile	680	276532	2.46E-03
c.557C>T	p.Pro186Leu	11	243662	4.51E-05
c.530G>A	p.Arg177His	321	267214	1.20E-03
c.529C>T	p.Arg177Cys	17	265018	6.41E-05
c.503T>C	p.Val168Ala	12	168932	7.10E-05
c.502G>A	p.Val168Met	53	168194	3.15E-04
c.495G>C	p.Glu165Asp	18	201294	8.94E-05
c.478C>T	p.Arg160Trp	297	204128	1.45E-03
c.472G>A	p.Val158Met	13652	206100	6.62E-02
c.461T>C	p.Ile154Thr	20	206608	9.68E-05
c.442G>A	p.Gly148Arg	13	200668	6.48E-05
c.440C>T	p.Pro147Leu	118	200128	5.90E-04

c.412A>G	p.Ser138Gly	10	159806	6.26E-05
c.362C>T	p.Pro121Leu	12	194964	6.15E-05
c.323C>T	p.Pro108Leu	6	129108	4.65E-05
c.187C>T	p.Arg63Trp	2	31394	6.37E-05
c.184A>C	p.Thr62Pro	39	259624	1.50E-04
c.131G>A	p.Arg44His	14	271948	5.15E-05
c.104G>A	p.Arg35Gln	16	241052	6.64E-05
c.94G>A	p.Glu32Lys	43	272046	1.58E-04
c.82G>A	p.Val28Met	41	270500	1.52E-04
c.74G>A	p.Ser25Asn	17	269206	6.31E-05
c.50G>A	p.Arg17Gln	20	235814	8.48E-05
c.13G>C	p.Gly5Arg	84	263208	3.19E-04

Supplemental Table 4. *MYBPC3* Variants in gnomAD with Allele Frequency >4E-05 (excluding P/LP variants from Supplemental Table 2)

Sample	Variant Location	Forward Primer (5' to 3')	Reverse Primer (5' to 3')	Wt (Bp)	If Exon excluded (bp)
**HCM 51**	Intron 30 acceptor	Exon 29	Exon 31	470	330
		GGCCTCAGGTGACCTGG	AGACGGGCTCCTTGGTGGTG		
**HCM 32, 64, 204**	Intron 30 donor	Exon 29	Exon 31	470	330
		GGCCTCAGGTGACCTGG	AGACGGGCTCCTTGGTGGTG		
**HCM 213**	Intron 12 acceptor	Exon 6	Exon 15	667	503
		CGAGCTGCACATCACCGATGCCC	GCTACACTTCTCGCCACCCACCACGC		
**HCM 114**	Intron 22 acceptor	Exon 21	Exon 24	422	341
		GCCCAGGCCGCATACCAGACAC	CCAGGATGGGCTGCCCGC		
**HCM 205**	Intron 22 Donor	Exon 21	Exon 24	422	341
		GCCCAGGCCGCATACCAGACAC	CCAGGATGGGCTGCCCGC		

Supplemental Table 5. Primers to identify exon skipping at splice sites.

Mutation	Mutation Location	Forward Primer (5' to 3')	Reverse Primer (5' to 3')	If Intron included (bp)	If DNA Contamination (bp)
**HCM 51**	Intron 30 acceptor	Intron 30*	Exon 32	398	509
		TAGGCCCGGCAGACCCAGG	GCTTGGGGCCTCGGAGAAGTCC		
**HCM 32, 64, 204**	Intron 30 donor	Intron 30*	Exon 32	398	509
		TAGGCCCGGCAGACCCAGG	GCTTGGGGCCTCGGAGAAGTCC		
**HCM 213**	Intron 12 acceptor	Intron 12	Exon 15	362	705
		CGGGGCGGCACAGAGGGGATTG	GCTACACTTCTCGCCACCCACCACGC		
**HCM 114**	Intron 22 acceptor	Intron 22	Exon 24***	588	1313
		GCCGGGGTCTTGCTCCTGCCTG	CCAGGATGGGCTGCCCGC		
**HCM 205**	Intron 22 donor	Intron 22	Exon 24***	588	1313
		GCCGGGGTCTTGCTCCTGCCTG	CCAGGATGGGCTGCCCGC		

Supplemental Table 6. Primers to identify intron inclusion at splice sites.

Supplemental Material References

1. Ho CY, Day SM, Ashley EA, Michels M, Pereira AC, Jacoby D, Cirino AL, Fox JC, Lakdawala NK, Ware JS, et al. Genotype and Lifetime Burden of Disease in Hypertrophic Cardiomyopathy: Insights from the Sarcomeric Human Cardiomyopathy Registry (SHaRe). *Circulation*. 2018;138:1387-1398. doi: 10.1161/CIRCULATIONAHA.117.033200. Epub 2018 Aug 23.
2. Richards S, Aziz N, Bale S, Bick D, Das S, Gastier-Foster J, Grody WW, Hegde M, Lyon E, Spector E, et al. Standards and guidelines for the interpretation of sequence variants: a joint consensus recommendation of the American College of Medical Genetics and Genomics and the Association for Molecular Pathology. *Genet Med*. 2015;17:405-24. doi: 10.1038/gim.2015.30. Epub 2015 Mar 5.
3. Kelly MA, Caleshu C, Morales A, Buchan J, Wolf Z, Harrison SM, Cook S, Dillon MW, Garcia J, Haverfield E, et al. Adaptation and validation of the ACMG/AMP variant classification framework for MYH7-associated inherited cardiomyopathies: recommendations by ClinGen's Inherited Cardiomyopathy Expert Panel. *Genet Med*. 2018;20:351-359. doi: 10.1038/gim.2017.218. Epub 2018 Jan 4.
4. Karczewski KJ, Francioli LC, Tiao G, Cummings BB, Alföldi J, Wang Q, Collins RL, Laricchia KM, Ganna A, Birnbaum DP, et al. Variation across 141,456 human exomes and genomes reveals the spectrum of loss-of-function intolerance across human protein-coding genes. 2019.
5. Helms AS, Davis FM, Coleman D, Bartolone SN, Glazier AA, Pagani F, Yob JM, Sadayappan S, Pedersen E, Lyons R, et al. Sarcomere mutation-specific expression patterns in human hypertrophic cardiomyopathy. *Circulation Cardiovascular genetics*. 2014;7:434-43.
6. Singer ES, Ingles J, Semsarian C and Bagnall RD. Key Value of RNA Analysis of MYBPC3 Splice-Site Variants in Hypertrophic Cardiomyopathy. *Circ Genom Precis Med*. 2019;12:e002368. doi: 10.1161/CIRCGEN.118.002368.
7. Ito K, Patel PN, Gorham JM, McDonough B, DePalma SR, Adler EE, Lam L, MacRae CA, Mohiuddin SM, Fatkin D, et al. Identification of pathogenic gene mutations in LMNA and MYBPC3 that alter RNA splicing. *Proc Natl Acad Sci U S A*. 2017;114:7689-7694. doi: 10.1073/pnas.1707741114. Epub 2017 Jul 5.
8. Walsh R, Mazzarotto F, Whiffin N, Buchan R, Midwinter W, Wilk A, Li N, Felkin L, Ingold N, Govind R, et al. Quantitative approaches to variant classification increase the yield and precision of genetic testing in Mendelian diseases: the case of hypertrophic cardiomyopathy. *Genome Med*. 2019;11:5. doi: 10.1186/s13073-019-0616-z.
9. O'Mahony C, Jichi F, Pavlou M, Monserrat L, Anastasakis A, Rapezzi C, Biagini E, Gimeno JR, Limongelli G, McKenna WJ, et al. A novel clinical risk prediction model for sudden cardiac death in hypertrophic cardiomyopathy (HCM risk-SCD). *Eur Heart J*. 2014;35:2010-20. doi: 10.1093/eurheartj/eht439. Epub 2013 Oct 14.
10. Nistri S, Olivotto I, Betocchi S, Losi MA, Valsecchi G, Pinamonti B, Conte MR, Casazza F, Galderisi M, Maron BJ, et al. Prognostic significance of left atrial size in patients with hypertrophic cardiomyopathy (from the Italian Registry for Hypertrophic Cardiomyopathy). *Am J Cardiol*. 2006;98:960-5. doi: 10.1016/j.amjcard.2006.05.013. Epub 2006 Aug 14.
11. Glazier AA, Hafeez N, Mellacheruvu D, Basrur V, Nesvizhskii AI, Lee LM, Shao H, Tang V, Yob JM, Gestwicki JE, et al. HSC70 is a chaperone for wild-type and mutant cardiac myosin binding protein C. *JCI Insight*. 2018;3(11).99319. doi: 10.1172/jci.insight.99319. eCollection 2018 Jun 7.
12. Bray MA, Sheehy SP and Parker KK. Sarcomere alignment is regulated by myocyte shape. *Cell motility and the cytoskeleton*. 2008;65:641-51.
