# Supplementary material for: Spatial and Functional Distribution of MYBPC3 Pathogenic Variants and Clinical Outcomes in Patients With Hypertrophic Cardiomyopathy
Source: Circ Genom Precis Med. 2020 Aug 25;13(5):396–405. doi: 10.1161/CIRCGEN.120.002929 (PMC7676622; doi:10.1161/CIRCGEN.120.002929)
Supplement: Supplementary file 2 [file hcg-13-396-s002.pdf]

Spatial and Functional Distribution of *MYBPC3* Pathogenic Variants and Clinical Outcomes in Patients with Hypertrophic Cardiomyopathy

Running title: *Helms et al.; MYBPC3 Variants in Hypertrophic Cardiomyopathy*

Adam S. Helms, MD, MS[1]; Andrea D. Thompson, MD, PhD[1]; Amelia A. Glazier, PhD[2]; Neha Hafeez, BS[1]; Samat Kabani, MD[1]; Juliani Rodriguez, BS[1]; Jaime M. Yob, MS[1]; Helen Woolcock[1]; Francesco Mazzarotto, PhD[3,4]; Neal K. Lakdawala, MD[5]; Samuel G. Wittekind, MD, MSc[6]; Alexandre C. Pereira, MD, PhD[7]; Daniel L. Jacoby, MD[8]; Steven D. Colan, MD[9]; Euan A. Ashley, MRCP, Dphil[10]; Sara Saberi, MD, MS[1]; James S. Ware, PhD, MRCP[4]; Jodie Ingles, PhD, MPH[11]; Christopher Semsarian, MBBS, PhD[11]; Michelle Michels, MD, PhD[12]; Iacopo Olivotto, MD[3,13]; Carolyn Y. Ho, MD[5]; Sharlene M. Day, MD[14]

Circulation: Genomic

[1]Cardiovascular Medicine, [2]Molecular & Integrative Physiology, Univ of Michigan, Ann Arbor, MI; [3]Dept of Experimental & Clinical Medicine, University of Florence, Florence, Italy; [4]National Heart & Lung Inst & Royal Brompton Cardiovascular Rsrch Ctr, Imperial College London, London, UK; [5]Cardiovascular Medicine, Brigham & Women's Hospital, Harvard Medical School, Boston, MA; [6]Cincinnati Children's Hospital Medical Ctr, Heart Inst, Cincinnati, OH; [7]Heart Inst (InCor), Univ of Sao Paolo Medical School, Sao Paulo, Brazil; [8]Cardiovascular Medicine, Yale Univ, New Haven, CT; [9]Dept of Cardiology, Boston Children's Hospital, Boston, MA; [10]Center for Inherited Heart Disease, Stanford Univ, Stanford, CA; [11]Agnes Ginges Centre for Molecular Cardiology at Centenary Institute, The Univ of Sydney, Australia; [12]Dept of Cardiology, Erasmus Medical Ctr, Rotterdam, the Netherlands; [13]Cardiomyopathy Unit, Careggi Univ Hospital, Florence, Italy; [14]Cardiovascular Medicine, Univ of Pennsylvania, Philadelphia, PA

Correspondence:

Adam S. Helms, MD, MS
Cardiovascular Division
University of Michigan
1150 W. Medical Center Dr.
Ann Arbor, MI 48109
E-mail: adamhelm@umich.edu

Sharlene M. Day, MD
Cardiovascular Division
University of Pennsylvania
3400 Civic Center Blvd.
Philadelphia, PA 19104
Email: sharlene.day@pennmedicine.upenn.edu

Journal Subject Terms: Genetics; Cardiomyopathy; Hypertrophy

Abstract:

Background - Pathogenic variants in *MYBPC3*, encoding cardiac MyBP-C, are the most common cause of familial hypertrophic cardiomyopathy. A large number of unique *MYBPC3* variants and relatively small genotyped HCM cohorts have precluded detailed genotype-phenotype correlations.

Methods - Patients with HCM and *MYBPC3* variants were identified from the Sarcomeric Human Cardiomyopathy Registry (SHaRe). Variant types and locations were analyzed, morphologic severity was assessed, and time-event analysis was performed (composite clinical outcome of sudden death, class III/IV heart failure, LVAD/transplant, atrial fibrillation). For selected missense variants falling in enriched domains, myofilament localization and degradation rates were measured *in vitro*.

Results - Among 4,756 genotyped HCM patients in SHaRe, 1,316 patients were identified with adjudicated pathogenic truncating (N=234 unique variants, 1047 patients) or non-truncating (N=22 unique variants, 191 patients) variants in *MYBPC3*. Truncating variants were evenly dispersed throughout the gene, and hypertrophy severity and outcomes were not associated with variant location (grouped by 5' - 3' quartiles or by founder variant subgroup). Non-truncating pathogenic variants clustered in the C3, C6, and C10 domains (18 of 22, 82%, p<0.001 vs. gnomAD common variants) and were associated with similar hypertrophy severity and adverse event rates as observed with truncating variants. MyBP-C with variants in the C3, C6, and C10 domains was expressed in rat ventricular myocytes. C10 mutant MyBP-C failed to incorporate into myofilaments and degradation rates were accelerated by ~90%, while C3 and C6 mutant MyBP-C incorporated normally with degradation rate similar to wild-type.

Conclusions - Truncating variants account for 91% of *MYBPC3* pathogenic variants and cause similar clinical severity and outcomes regardless of location, consistent with locus-independent loss-of-function. Non-truncating *MYBPC3* pathogenic variants are regionally clustered, and a subset also cause loss-of-function through failure of myofilament incorporation and rapid degradation. Cardiac morphology and clinical outcomes are similar in patients with truncating vs. non-truncating variants.

Key words: hypertrophic cardiomyopathy; myosin binding protein; sarcomere; *MYBPC3*

American Heart Association.
Circulation: Genomic and Precision Medicine

Nonstandard Abbreviations and Acronyms:

ACMG: American College of Medical Genetics and Genomics

AMP: Association for Molecular Pathology

gnomAD: Genome Aggregation Database

HCM: Hypertrophic cardiomyopathy

LV: Left ventricle

LVAD: Left ventricular assist device

MyBP-C: Myosin binding protein C

NMD: Nonsense mediated RNA decay

NRVM: Neonatal rat ventricular myocytes

PTC: premature termination codon

SHaRe: Sarcomeric Human Cardiomyopathy Registry

Circulation: Genomic and Precision Medicine

Introduction

Familial hypertrophic cardiomyopathy (HCM) is an autosomal dominant condition, and pathogenic variants in cardiac myosin binding protein C (protein abbreviation, MyBP-C, encoded by the gene, *MYBPC3*) are the most common cause.[1] MyBP-C is a sarcomeric protein that binds both actin and myosin and regulates cardiac contractility by modulating myofilament sliding velocity.[2, 3] Because a large number of unique *MYBPC3* variants have been associated with HCM, small, single-center cohorts have had limited capacity to systematically analyze genotype-phenotype relationships, particularly given the marked variability in penetrance of *MYBPC3*-associated HCM.[4-7] Resolving these gaps in knowledge will be critical to further personalized risk assessment and management of patients with HCM.

Most *MYBPC3* pathogenic variants are frameshift, nonsense, or splice-site variants that result in premature termination codons (PTCs). PTC-containing transcripts are targeted for degradation through nonsense mediated RNA decay (NMD), and hence may cause disease through allelic loss of function (resulting in reduced levels of MyBP-C). Consistent with allelic insufficiency, we and others have shown a ~40% reduction in MyBP-C in heart tissue from HCM patients,[8,9] due to a rate-limiting reduction in *MYBPC3* mRNA.[10] These studies support the hypothesis that truncating variants in *MYBPC3* likely exert a similar primary effect, independent of the specific variant locus. However, comparative analyses across the full genotypic and phenotypic spectrum of truncating variants have not been possible due to the small size of previously-available cohorts. Distinct from truncating *MYBPC3* variants, non-truncating pathogenic variants (including missense and short in-frame deletions/insertion variants) account for ~15% of *MYBPC3* HCM. The mechanism(s) of *MYBPC3* non-truncating pathogenic variants are largely unknown, and it is unclear whether phenotypic expression or clinical outcomes are different in patients carrying missense variants.[7, 11] A greater understanding of the disease-causing mechanism(s) of non-truncating *MYBPC3* pathogenic variants through functional analyses could improve adjudication of variant pathogenicity and expand the pool of clinically actionable gene test results.

American Heart Association.
Circulation: Genomic and Precision Medicine

Here, we utilize the largest registry of combined genetics and clinical data for HCM to date, the Sarcomeric Human Cardiomyopathy Registry[1] (SHaRe), to generate an adjudicated and comprehensive compendium of *MYBPC3* variation, analyze regional variation within *MYBPC3*, and correlate clinical phenotypes. We find that pathogenic truncating variants are homogeneously distributed throughout the gene, in contrast to non-truncating *MYBPC3* pathogenic variants that cluster in specific protein domains. Disease severity is highly variable

in *MYBPC3* HCM, and we show that this variability is largely independent of variant location or the specific truncating or non-truncating variant based on both disease severity metrics and clinical outcomes. Finally, we experimentally test functional effects of non-truncating pathogenic variants in the identified variant-enriched domains and identify a subset that exhibit allelic loss of function.

Methods

The methods used are described for purposes of replicating the study procedure. Individual patient data will not be made available for purposes of reproducing the results. The study was independently approved by the institutional review board at each center. A detailed methods section is available in the Supplemental Data.

American Heart Association.
Circulation: Genomic and Precision Medicine

Results

Clinical Profile of *MYBPC3* Mutation HCM

Among 4,756 genotyped patients with a clinical diagnosis of HCM in SHaRe at the time of this study, 2,179 (46%) were found to have pathogenic sarcomere gene variants, and, of this sarcomere-positive group, 1,316 (60%) were identified with adjudicated pathogenic *MYBPC3* variants. 1238 (94%) of the *MYBPC3* group carried single *MYBPC3* pathogenic variants without pathogenic variants in other sarcomere genes and comprised the primary study group. The largest subset of these patients (N=1047, 71%) carried truncating *MYBPC3* variants (234 unique variants), while 29% (N=191) had non-truncating variants (22 unique variants).

The demographic and clinical profile of patients with *MYBPC3* pathogenic variants is shown in Table 1. The majority (76%) of patients presented in early-mid adulthood (age 18-60

years) with a minority of pediatric (13%) or late adulthood (10%) presentations. The average age of diagnosis was younger among patients with non-truncating pathogenic variants due to a greater percentage with pediatric diagnoses (24% vs. 11%, p=0.0001). Maximum left ventricular (LV) wall thickness was greater in the relatively small subset of pediatric patients with non-truncating variants, but was similar in other age groups. LV ejection fraction was similarly elevated at a young age in both groups and declined similarly in later age groups. Left atrial diameter progressively increased to a similar extent with increasing age in both truncating and non-truncating groups (with the single exception of the smaller sized group of non-truncating variant patients at age >60; N=17). The distributions of maximum wall thickness, left atrial size, and age of diagnosis among non-truncating and truncating pathogenic variant cases are shown in Figure 1A-C. Time to event analysis for composite adverse outcomes revealed no difference between patients with non-truncating or truncating pathogenic variants (Figure 1D), and this result was not different when including probands only (Supplemental Figure 1A). There were similarly no differences between the groups for heart failure or ventricular arrhythmia composite outcomes (not shown).

Circulation: Genomic and Precision Medicine

Morphologic Severity and Adverse Events Are Similar Across Truncating *MYBPC3* Pathogenic Variants

If truncating *MYBPC3* variants cause allelic insufficiency as their primary consequence, then the location of the variant within the gene would not be expected to influence the disease severity. To test this, we categorized truncating *MYBPC3* pathogenic variants into quartiles by 5' to 3' location and compared morphologic markers of severity and adverse outcomes. We found no statistically significant difference in maximum wall thickness or age-adjusted left atrial diameter

among these groups (Figure 2A-B). Composite adverse events were also similar when stratified by variant location quartile (Figure 2C) or by truncating variant type (Supplemental Figure 1B).

Morphologic Severity, Adverse Events, and Variability in Phenotype Are Similar Among Founder and Non-Founder Truncating *MYBPC3* Pathogenic Variants

Several founder truncating pathogenic variants in *MYBPC3* have a high prevalence among HCM patients. In SHaRe, 4 distinct founder truncating variants exist in large numbers, enabling comparison across subgroups that share the same primary causative sarcomere gene mutation. These founder populations consisted of 142 individuals with the c.742G>A variant (exonic splice variant causing exon skipping and PTC[12]), 143 with the c.2373insG variant (insertion variant causing frameshift and PTC)[13], 67 with the c.2827C>T variant (nonsense variant), and 58 with the c.2864_2865del variant (deletion causing frameshift and PTC). Left ventricular hypertrophy was similar across each of these 4 founder populations and the remaining non-founder truncating variant patients (N=638), further supporting that different truncating variants exert a similar effect (Figure 2D). Additionally, adverse events were similar in each founder population compared to patients with non-founder truncating variants (Figure 2E).

American Heart Association.
Circulation: Genomic and Precision Medicine

HCM is known to have broad variance in phenotypic severity across individuals. This variance in expressivity has been thought to be due to heterogeneity of effect size of underlying pathogenic variants, the influence of background genetic variation (i.e. genetic modifiers), and clinical comorbidities.[14-17] Taking advantage of the founder populations, we compared variances across these subgroups each carrying identical pathogenic variants. As shown in the histogram plot of maximum wall thickness in Figure 2F, the 4 founder populations demonstrate similar variance (mean of standard deviations 5.96 $\pm$ 0.79 mm) compared to the remainder of the truncating variant population (standard deviation 5.98 mm, p=NS). Taken together, these

findings indicate that truncating variants likely exert a similar primary effect, and the marked variance in disease phenotype among truncating variant patients is caused by additional genetic and non-genetic factors, independent of the driving *MYBP3* variant.

Variant Classification and Distribution of Truncating and Non-Truncating *MYBP3* Pathogenic Variants in HCM

MYBPC3 truncating variant types in SHaRe patients consisted of 110 unique insertion/deletion variants, 55 unique nonsense variants, and 69 unique splice variants (Supplemental Table 1). Classification of potential splice variants is complicated by the fact that only a portion of splice consensus sites are strictly conserved. The 69 unique splice pathogenic variants were classified through application of the American College of Medical Genetics (ACMG) and Genomics Association for Molecular Pathology (AMP) criteria, combined with enrichment in SHaRe, prior experimental confirmation, and independent experimental confirmation in select cases (Supplemental Tables 1-2, Supplemental Figure 2). These criteria left a total of 26 of 99 potential intronic variants classified at VUS status. Six exonic splice variants were identified at the last base pair position in their respective exons (donor -1 position), 4 of which have had prior experimental confirmation of splice disruption in human heart tissue.[12, 18] These splice variants (c.655G>C, c.772G>A, c.772G>C, c.1090G>A, c.1624G>C, c.1790G>A) were consequently classified as truncating – an important distinction since erroneous classification as missense variants would impact clustering analysis of the non-truncating variants. Comparison of our clinical-genetics assignment of variant pathogenicity to the *MYBPC3* splice variant prediction mini-gene splice assay developed by Ito, et al[19] demonstrated a high, though not perfect, level of concordance, with 20 out of 23 variants (87%) in agreement (Supplemental Tables 1 and 3). Non-truncating pathogenic variants were less common than truncating variants, with only 22

American Heart Association.
Circulation: Genomic and Precision Medicine

unique variants meeting criteria for pathogenicity, present in a total of 191 patients carrying a single sarcomere gene pathogenic variant (15% of *MYBPC3* pathogenic variant patients). The potential pathogenicity of 147 non-truncating VUS's could not be resolved with clinical data from SHaRe.

To determine regional variation in the distribution of *MYBPC3* pathogenic variants, we mapped all unique *MYBPC3* pathogenic variants in SHaRe by location within the coding sequence, stratified by truncating or non-truncating variant type (Figure 3). Truncating variants were dispersed throughout the coding regions of the gene without evidence of regional clustering. In addition, unique truncating variants were similarly prevalent in the N-terminus (including a variant that disrupts the start codon). In contrast, non-truncating pathogenic variants were primarily localized in the C3, C6, and C10 domains (18 of 22, 82%) – as compared to non-truncating common variants in the Genome Aggregation Database (gnomAD) that were distributed throughout the gene (Figure 1, Supplemental Table 3-4). The C3 domain alone accounted for most individuals with *MYBPC3* non-truncating variants in SHaRe (177 of 191, 93%). Among gnomAD common variants, a lower percentage (17%, 23 of 135) localized to the C3, C6, or C10 domains (p<0.0001 compared to SHaRe).

American Heart Association.
Circulation: Genomic and Precision Medicine

Experimental Confirmation of Domain Specific Effects of MyBP-C Non-Truncating Pathogenic variants on Myofilament Incorporation and Degradation Rate

While strong evidence supports allelic insufficiency is the primary mechanism across the spectrum of truncating *MYBPC3* variants, the mechanism(s) of non-truncating *MYBPC3* pathogenic variants has not been resolved. We hypothesized that some non-truncating *MYBPC3* pathogenic variants may also cause loss of function, but through lack of normal protein localization or structural stability rather than reduced expression. Therefore, we first tested

whether exogenously expressed MyBP-C with non-truncating pathogenic variants incorporates normally into the myofilaments. We expressed FLAG-epitope labeled MyBP-C with or without pathogenic non-truncating variants in neonatal rat ventricular myocytes (NRVMs) and analyzed localization by immunofluorescence. We found that MyBP-C containing representative C3 or C6 domain non-truncating variants localized normally to the sarcomere A bands while MyBP-C containing C10 domain non-truncating variants was essentially absent from the myofilaments (Figure 4).

A lack of mutant MyBP-C myofilament incorporation could be either due to perturbation of binding sites required for correct localization or protein instability. To determine if pathogenic variants in the C10 domain result in protein destabilization, we performed cyclohexamide pulse-chase experiments using NRVMs transduced with FLAG-tagged mutant MyBP-C for representative variants. Consistent with MyBP-C destabilization as a consequence of pathogenic variants in the C10 domain, we found a marked 90% reduction in protein half-life (Figure 5, Table 2). In contrast, most pathogenic variants in the C3 and C6 domains resulted in MyBP-C protein half-lives that were not significantly different from wild-type MyBP-C, though the Arg502Trp variant resulted in a modest 36% shorter protein half-life compared to wild-type ($p=0.04$). Paradoxically, the Arg810His variant resulted in a 44% prolonged MyBP-C protein half-life compared to wild-type ($P=0.008$).

Circulation: Genomic and Precision Medicine

Discussion

Despite genetic variants in *MYBPC3* being the most common cause of familial HCM, identifying genotype-phenotype correlations has been elusive, due to the large number of individual pathogenic variants and small numbers of patients previously available to study from single

centers. Here, we harness the largest cohort of genotyped HCM patients to comprehensively describe *MYBPC3* genetic variation and associated clinical phenotypes.

A convergent theory of allelic insufficiency from truncating *MYBPC3* variants has emerged from human tissue, rodent, and iPSC model systems.[8, 10, 20-22] Reduction in MyBP-C relative to myosin alters sliding velocities as actin-myosin sliding reaches the C-zones, where MyBP-C is specifically present, resulting in a more rapid contractile deceleration toward peak force development.[2, 8, 10, 23] However, clinical-genetics data to confirm this theory have been notably absent. Our findings of a homogeneous distribution of HCM-causing truncating variants throughout *MYBPC3*, similar phenotypic severity across spatial quartiles in the coding sequence, and similar adverse event rates support the theory that disease results from a biologically similar loss of function mechanism across truncating variants, as opposed to dominant negative consequences from truncated MyBP-C protein (which has not been detectable in human heart or cellular models[9, 10, 12]). Furthermore, we found that 4 founder populations, with distinct truncating variants and sizable numbers in SHaRe, exhibit similar disease severity and adverse event rates as compared to non-founder truncating variant patients. This result extends findings from a single site investigation of the Netherlands founder cohort[24], and counters smaller series that have suggested less pathogenic effects in truncating variant founder cohorts.[25, 26] A major implication of these results is that patients with truncating *MYBPC3* variants would likely derive similar benefit from targeted treatment approaches irrespective of the specific location of the truncating variant.

American Heart Association.
Circulation: Genomic and Precision Medicine

We further leveraged the truncating variant founder populations in SHaRe to investigate the variability in expressivity in HCM. HCM exhibits vast genetic and phenotypic heterogeneity, which has been a major challenge in determining genotype-phenotype relationships.[27] We found

that patients with founder variants had a similar distribution of phenotypic features and clinical outcomes as non-founder HCM patients with truncating variants. This finding suggests that the variability in disease phenotype among *MYBPC3* truncating variant carriers is not dictated solely by the primary pathogenic variant. An important implication of this finding is that additional genetic and non-genetic modifiers likely account for the broad variance in phenotypic severity among patients with *MYBPC3* HCM.

We also demonstrate that *MYBPC3* non-truncating pathogenic variants, accounting for 15% of *MYBPC3* pathogenic variants, generally had a similar phenotypic effect as truncating variants. Minor differences between the groups included a modestly greater proportion of pediatric diagnoses in the non-truncating group and modestly reduced prevalence of LVOT obstruction. However, maximal LV wall thickness across all other age groups, and adverse event rates were highly similar.

American Heart Association.
Circulation: Genomic and Precision Medicine

Because non-truncating variants are robustly adjudicated in SHaRe, we were able to identify strong evidence of domain clustering. We then demonstrated that a subgroup of non-truncating pathogenic variants (those in the C10 domain) renders the resultant mutant protein susceptible to rapid degradation, resulting in a loss of function mechanism similar to truncating variants. In contrast, we show no destabilization in the majority of C3 and C6 domain mutant proteins, which integrate normally in myofilaments. The C3 variant Arg502Trp alters the electrostatic properties of the domain, but how this alteration affects MyBP-C function is not known.[28] In engineered heart tissue, overexpression of the C3 mutant Gly531Arg (not present in SHaRe), caused hypercontractility at low calcium levels and was not able to rescue MyBP-C knock-out tissues.[29] Further study is required to fully elucidate the impact of C3 and C6 pathogenic variants on contractile function.

In contrast to the clustering evident for pathogenic non-truncating variants, VUS's in *MYBPC3* were relatively common in the SHaRe cohort (N=148, 87% of all unique *MYBPC3* non-truncating variants). Accurate prediction of pathogenicity of sarcomere VUS's is a major challenge for interpretation of genetic testing results and determination of the suitability for cascade testing in family members. Although we confirmed enrichment of non-truncating pathogenic variants in specific MyBP-C domains, as also shown in an independent cohort by Walsh and colleagues[30], the presence of common variants in gnomAD in these same domains should preclude a complete reliance on a generalized domain-centric approach to determine variant pathogenicity. Nevertheless, the presence of a variant in the C3, C6, or C10 domains in a patient with HCM increases the probability of pathogenicity, and could be used as a supportive criterion with other clinical variables in variant classification. Moreover, identifying VUS's that cause protein instability could be a useful strategy for functional annotation of variants.

Circulation: Genomic and Precision Medicine

Several limitations to our study should be considered. This was a retrospective, observational study. Although we analyzed by far the largest cohort of HCM patients with *MYBPC3* pathogenic variants to date, the study may be underpowered to detect small differences in phenotype severity or adverse events between groups. In addition, we analyzed pathogenic variant carriers in groups based on variant type and location, but further subdivision to individual pathogenic variants was only feasible for the founder subpopulations. As such, differences in effect size for specific pathogenic variants, particularly in the case of the non-truncating variants, could still exist. Both the SHaRe population and gnomAD populations predominantly consist of individuals from European ancestry. Although these attributes lend confidence to the calculation of the odds ratios for HCM-associated versus common population variants reported here, the results are not necessarily representative of genetic variation in other ancestries. Relatedly, the

SHaRe population has a greater proportion of patients with HCM with truncating founder variants due to inclusion of certain European sites (The Netherlands, Italy). Lastly, we strategically focused experimental testing of non-truncating pathogenic variants to the impact on protein stability and only examined a subset of representative variants. Future work will be needed to further resolve the functional effects of pathogenic non-truncating *MYBPC3* variants that do not destabilize the protein structure and extending these analyses more comprehensively across *MYBPC3* non-truncating variants.

In conclusion, we leverage the largest cohort of patients with *MYBPC3* pathogenic variants to date to develop a compendium of benign, pathogenic, and uncertain *MYBPC3* variants and identify genotype-phenotype correlations. Our results demonstrate that phenotypic severity and clinical outcomes are similar across the range of *MYBPC3* pathogenic variant carriers, without obvious associations based on the location of truncating variants, founder or non-founder truncating variant carriers, or truncating versus non-truncating variants. These findings highlight the need to identify additional background genetic and non-genetic modifiers that influence the broadly variable HCM disease phenotype. In addition, we show that non-truncating pathogenic variants cluster in particular MyBP-C domains, with those variants in the C10 domain exhibiting protein destabilization leading to loss of function, in contrast to a second subset exhibiting normal myofilament incorporation and stability.

American Heart Association.
Circulation: Genomic and Precision Medicine

Sources of Funding: Funding for SHaRe has been provided through an unrestricted research grant from Myokardia, Inc, a startup company that is developing therapeutics that target the sarcomere. MyoKardia, Inc, had no role in approving the content of this manuscript. Dr. Helms is supported by funding from the National Institutes of Health (K08HL130455). Dr. Thompson is supported by the National Institutes of Health (T32 HL007853). Dr Ware is supported by the Wellcome Trust (107469/Z/15/Z) and the Medical Research Council (United Kingdom). Dr

Ingles a recipient of a National Health and Medical Research Council (NHMRC) Career Development Fellowship (#1162929). Dr Semsarian is the recipient of a NHMRC Practitioner Fellowship (#1059156). Dr Olivotto is supported by the Italian Ministry of Health ("Left Ventricular Hypertrophy in Aortic Valve Disease and Hypertrophic Cardiomyopathy: Genetic Basis, Biophysical Correlates and Viral Therapy Models" [RF-2013-02356787] and NET-2011-02347173 [Mechanisms and Treatment of Coronary Microvascular Dysfunction in Patients sith Genetic or Secondary Left Ventricular Hypertrophy]) and by the Tuscany Registry of Sudden Cardiac Death (ToRSADE) project (FAS-Salute 2014, Regione Toscana). Dr Ho is supported by funding from the National Institutes of Health (1P50HL112349 and 1U01HL117006). Dr Day is supported by funding from the National Institutes of Health (R01 11572784), the American Heart Association (grant in aid), the Children's Cardiomyopathy Foundation, and the Protein Folding Disease Initiative (University of Michigan).

Disclosures: Drs. Helms, Ho, Day, Saberi, Olivotto, Colan, Ingles and Ashley receive research support from MyoKardia, Inc. The other authors report no relevant conflicts of interest.

Circulation: Genomic and Precision Medicine

References:

1. Ho CY, Day SM, Ashley EA, Michels M, Pereira AC, Jacoby D, Cirino AL, Fox JC, Lakdawala NK, Ware JS, et al. Genotype and Lifetime Burden of Disease in Hypertrophic Cardiomyopathy: Insights from the Sarcomeric Human Cardiomyopathy Registry (SHaRe). *Circulation*. 2018;138:1387-1398. doi: 10.1161/CIRCULATIONAHA.117.033200. Epub 2018 Aug 23.

2. Previs MJ, Beck Previs S, Gulick J, Robbins J, Warshaw DM. Molecular mechanics of cardiac myosin-binding protein C in native thick filaments. *Science*. 2012;337:1215-8. doi: 10.1126/science.1223602. Epub 2012 Aug 23.

3. Previs MJ, Prosser BL, Mun JY, Previs SB, Gulick J, Lee K, Robbins J, Craig R, Lederer WJ, Warshaw DM. Myosin-binding protein C corrects an intrinsic inhomogeneity in cardiac excitation-contraction coupling. *Sci Adv*. 2015;1:e1400205.

4. Van Driest SL, Vasile VC, Ommen SR, Will ML, Tajik AJ, Gersh BJ, Ackerman MJ. Myosin binding protein C mutations and compound heterozygosity in hypertrophic cardiomyopathy. *J Am Coll Cardiol*. 2004;44:1903-10. doi: 10.1016/j.jacc.2004.07.045.

5. Alfares AA, Kelly MA, McDermott G, Funke BH, Lebo MS, Baxter SB, Shen J, McLaughlin HM, Clark EH, Babb LJ, et al. Results of clinical genetic testing of 2,912 probands with

hypertrophic cardiomyopathy: expanded panels offer limited additional sensitivity. *Genet Med*. 2015;17:880-8. doi: 10.1038/gim.2014.205. Epub 2015 Jan 22.

6. Carrier L, Mearini G, Stathopoulou K, Cuello F. Cardiac myosin-binding protein C (MYBPC3) in cardiac pathophysiology. *Gene*. 2015;573:188-97. doi: 10.1016/j.gene.2015.09.008. Epub 2015 Sep 8.

7. Page SP, Kounas S, Syrris P, Christiansen M, Frank-Hansen R, Andersen PS, Elliott PM, McKenna WJ. Cardiac myosin binding protein-C mutations in families with hypertrophic cardiomyopathy: disease expression in relation to age, gender, and long term outcome. *Circ Cardiovasc Genet*. 2012;5:156-66. doi: 10.1161/CIRCGENETICS.111.960831. Epub 2012 Jan 20.

8. O'Leary TS, Snyder J, Sadayappan S, Day SM, Previs MJ. MYBPC3 truncation mutations enhance actomyosin contractile mechanics in human hypertrophic cardiomyopathy. *J Mol Cell Cardiol*. 2019;127:165-173.:10.1016/j.yjmcc.2018.12.003. Epub 2018 Dec 11.

9. Marston S, Copeland O, Jacques A, Livesey K, Tsang V, McKenna WJ, Jalilzadeh S, Carballo S, Redwood C, Watkins H. Evidence from human myectomy samples that MYBPC3 mutations cause hypertrophic cardiomyopathy through haploinsufficiency. *Circ Res*. 2009;105:219-22.

10. Helms AS, Tang VT, O'Leary TS, Friedline S, Wauchope M, Arora A, Wasserman AH, Smith ED, Lee LM, Wen X, et al. Effects of MYBPC3 loss of function mutations preceding hypertrophic cardiomyopathy. *JCI Insight*. 2019;26:133782.

11. Erdmann J, Raible J, Maki-Abadi J, Hummel M, Hammann J, Wollnik B, Frantz E, Fleck E, Hetzer R, Regitz-Zagrosek V. Spectrum of clinical phenotypes and gene variants in cardiac myosin-binding protein C mutation carriers with hypertrophic cardiomyopathy. *J Am Coll Cardiol*. 2001;38:322-30. doi: 10.1016/s0735-1097(01)01387-0.

12. Helms AS, Davis FM, Coleman D, Bartolone SN, Glazier AA, Pagani F, Yob JM, Sadayappan S, Pedersen E, Lyons R, et al. Sarcomere mutation-specific expression patterns in human hypertrophic cardiomyopathy. *Circ Cardiovasc Genet*. 2014;7:434-43.

13. Alders M, Jongbloed R, Deelen W, van den Wijngaard A, Doevendans P, Ten Cate F, Regitz-Zagrosek V, Vosberg HP, van Langen I, Wilde A, et al. The 2373insG mutation in the MYBPC3 gene is a founder mutation, which accounts for nearly one-fourth of the HCM cases in the Netherlands. *Eur Heart J*. 2003;24:1848-53. doi: 10.1016/s0195-668x(03)00466-4.

14. Daw EW, Chen SN, Czernuszewicz G, Lombardi R, Lu Y, Ma J, Roberts R, Shete S, Marian AJ. Genome-wide mapping of modifier chromosomal loci for human hypertrophic cardiomyopathy. *Hum Mol Genet*. 2007;16:2463-71.

15. Claes GR, van Tienen FH, Lindsey P, Krapels IP, Helderman-van den Enden AT, Hoos MB, Barrois YE, Janssen JW, Paulussen AD, Sels JW, et al. Hypertrophic remodelling in cardiac

Circulation: Genomic and Precision Medicine

regulatory myosin light chain (MYL2) founder mutation carriers. *Eur Heart J*. 2016;37:1815-22. doi: 10.1093/eurheartj/ehv522. Epub 2015 Oct 24.

16. Helms AS, Day SM. Hypertrophic cardiomyopathy: single gene disease or complex trait? *Eur Heart J*. 2016;37:1823-5. doi: 10.1093/eurheartj/ehv562. Epub 2015 Oct 27.

17. Wooten EC, Hebl VB, Wolf MJ, Greytak SR, Orr NM, Draper I, Calvino JE, Kapur NK, Maron MS, Kullo IJ, et al. Formin homology 2 domain containing 3 variants associated with hypertrophic cardiomyopathy. *Circ Cardiovasc Genet*. 2013;6:10-8. doi: 10.1161/CIRCGENETICS.112.965277. Epub 2012 Dec 19.

18. Singer ES, Ingles J, Semsarian C, Bagnall RD. Key Value of RNA Analysis of MYBPC3 Splice-Site Variants in Hypertrophic Cardiomyopathy. *Circ Genom Precis Med*. 2019;12:e002368. doi: 10.1161/CIRCGEN.118.002368.

19. Ito K, Patel PN, Gorham JM, McDonough B, DePalma SR, Adler EE, Lam L, MacRae CA, Mohiuddin SM, Fatkin D, et al. Identification of pathogenic gene mutations in LMNA and MYBPC3 that alter RNA splicing. *Proc Natl Acad Sci U S A*. 2017;114:7689-7694. doi: 10.1073/pnas.1707741114. Epub 2017 Jul 5.

20. Toepfer CN, Wakimoto H, Garfinkel AC, McDonough B, Liao D, Jiang J, Tai AC, Gorham JM, Lunde IG, Lun M, et al. Hypertrophic cardiomyopathy mutations in MYBPC3 dysregulate myosin. *Sci Transl Med*. 2019;11:eaat1199. doi: 10.1126/scitranslmed.aat1199.

21. Giles J, Patel JR, Miller A, Iverson E, Fitzsimons D, Moss RL. Recovery of left ventricular function following in vivo reexpression of cardiac myosin binding protein C. *J Gen Physiol*. 2019;151:77-89. doi: 10.1085/jgp.201812238. Epub 2018 Dec 20.

22. Stohr A, Friedrich FW, Flenner F, Geertz B, Eder A, Schaaf S, Hirt MN, Uebeler J, Schlossarek S, Carrier L, et al. Contractile abnormalities and altered drug response in engineered heart tissue from Mybpc3-targeted knock-in mice. *J Mol Cell Cardiol*. 2013;63:189-98.:10.1016/j.yjmcc.2013.07.011. Epub 2013 Jul 26.

23. de Lange WJ, Grimes AC, Hegge LF, Ralphe JC. Ablation of cardiac myosin-binding protein-C accelerates contractile kinetics in engineered cardiac tissue. *J Gen Physiol*. 2013;141:73-84. doi: 10.1085/jgp.201210837.

24. van Velzen HG, Schinkel AFL, Oldenburg RA, van Slegtenhorst MA, Frohn-Mulder IME, van der Velden J, Michels M. Clinical Characteristics and Long-Term Outcome of Hypertrophic Cardiomyopathy in Individuals With a MYBPC3 (Myosin-Binding Protein C) Founder Mutation. *Circ Cardiovasc Genet*. 2017;10.CIRCGENETICS.116.001660. doi: 10.1161/CIRCGENETICS.116.001660.

25. Teirlinck CH, Senni F, Malti RE, Majoor-Krakauer D, Fellmann F, Millat G, Andre-Fouet X, Pernot F, Stumpf M, Boutarin J, et al. A human MYBPC3 mutation appearing about 10 centuries

American Heart Association.
Circulation: Genomic and Precision Medicine

ago results in a hypertrophic cardiomyopathy with delayed onset, moderate evolution but with a risk of sudden death. *BMC Med Genet*. 2012;13:105.:10.1186/1471-2350-13-105.

26. Jaaskelainen P, Miettinen R, Karkkainen P, Toivonen L, Laakso M, Kuusisto J. Genetics of hypertrophic cardiomyopathy in eastern Finland: few founder mutations with benign or intermediary phenotypes. *Ann Med*. 2004;36:23-32.

27. Ho CY, Charron P, Richard P, Girolami F, Van Spaendonck-Zwarts KY, Pinto Y. Genetic advances in sarcomeric cardiomyopathies: state of the art. *Cardiovasc Res*. 2015;105:397-408. doi: 10.1093/cvr/cvv025. Epub 2015 Jan 29.

28. Zhang XL, De S, McIntosh LP, Paetzel M. Structural characterization of the C3 domain of cardiac myosin binding protein C and its hypertrophic cardiomyopathy-related R502W mutant. *Biochemistry*. 2014;53:5332-42. doi: 10.1021/bi500784g. Epub 2014 Aug 7.

29. Wijnker PJ, Friedrich FW, Dutsch A, Reischmann S, Eder A, Mannhardt I, Mearini G, Eschenhagen T, van der Velden J, Carrier L. Comparison of the effects of a truncating and a missense MYBPC3 mutation on contractile parameters of engineered heart tissue. *J Mol Cell Cardiol*. 2016;97:82-92. 10.1016/j.yjmcc.2016.03.003. Epub 2016 Apr 22.

30. Walsh R, Mazzarotto F, Whiffin N, Buchan R, Midwinter W, Wilk A, Li N, Felkin L, Ingold N, Govind R, et al. Quantitative approaches to variant classification increase the yield and precision of genetic testing in Mendelian diseases: the case of hypertrophic cardiomyopathy. *Genome Med*. 2019;11:5. doi: 10.1186/s13073-019-0616-z.

American Heart Association.

Circulation: Genomic and Precision Medicine

Table 1. Demographic Characteristics of Patients with Truncating and Non-Truncating *MYBC3* Pathogenic variants. Morphologic parameters were obtained from first echocardiogram at a SHaRe site and stratified by age group at the time of the echocardiogram

	**Non-Truncating Pathogenic Variant (N=191)**	**Truncating Pathogenic Variant (N=1,047)**	**p-value**
Age at Diagnosis (years)	34.18 ± 17.98	38.96 ± 16.75	0.0004
Age Group at Diagnosis			0.0001
<18	44 (23.66%)	119 (11.41%)	
18-40	65 (34.95%)	409 (39.21%)	
40-60	60 (32.26%)	408 (39.12%)	
>60	17 (9.14%)	107 (10.26%)	
Proband	158 (82.7%)	814 (77.1%)	0.1
Female	74 (38.7%)	399 (37.8%)	0.86
Race			0.15
European Ancestry	166 (93.79%)	939 (92.60%)	
Asian Ancestry	2 (1.13%)	23 (2.27%)	
African Ancestry	1 (0.56%)	29 (2.86%)	
Other or Not Reported	6 (3.39%)	17 (1.68%)	
Family History of HCM	108 (56.54%)	590 (55.87%)	0.93
LV Maximum Wall Thickness			
Age <18	21.3 + 8.1	17.5 + 7.1	0.01
Age 18 – 40	21.2 + 6.2	21.3 + 6.1	1
Age 40-60	20.1 + 5.1	19.8 + 5.0	0.99
Age >60	18.3 + 4.9	19.4 + 4.9	0.77
Left Atrial Diameter			
Age <18	30.7 ± 7.6	33.5 ± 7.7	0.7
Age 18 – 40	38.9 ± 8.9	39.6 ± 11.7	0.87
Age 40-60	42.1 ± 11.1	42.8 ± 10.6	0.87
Age >60	36.8 ± 13.2	45.7 ± 12.7	0.004
LVOT Obstruction	36 (18.8%)	330 (31.5%)	0.004
LV Ejection Fraction			
Age <18	71.3 ± 6.2%	67.8 ± 7.6%	0.4
Age 18 – 40	62.2 ± 8.5%	63.1 ± 10.0%	0.95
Age 40-60	63.1 ± 11.4%	62.2 ± 9.8%	0.97
Age >60	61.8 ± 12.1%	61.2 ± 9.7%	0.99
Apical Variant	7 (3.7%)	26 (2.4%)	0.32

LV indicates left ventricular; LVOT indicates left ventricular outflow tract

Circulation: Genomic and Precision Medicine

Table 2. Non-Truncating Mutant MyBP-C Degradation Rates Measured by Cyclohexamide Pulse Chase

**Adenoviral Treatment**	**Half Life (hours)**	**95% Confidence Interval**	**p-value**
Wild-type control	5.06	4.15 - 6.50	--
Arg495Gln (C3)	5.50	3.95 - 9.06	0.72
Arg502Trp (C3)	3.24	2.51 - 4.56	0.044
Phe503Leu (C3)	5.82	4.34 - 8.81	0.51
Trp792Arg (C6)	3.41	2.66 - 5.04	0.083
Arg810His (C6)	8.92	6.46 - 14.40	0.008
Leu1238Pro (C10	0.27	0.16 - 0.88	P<0.0001
Gly1248-Cys1253 duplication (C10)	0.43	0.36 - 0.55	P<0.0001
Asn1257Lys (C10	0.29	0.25 - 0.35	P<0.0001

American Heart Association.
Circulation: Genomic and Precision Medicine

Figure Legends:

Figure 1. *MYBPC3* Non-Truncating Pathogenic Variants Cause Similar Phenotypic Severity and Adverse Event Rates as Truncating Variants. **A.** Distributions in maximum wall thickness demonstrate broad phenotypic variance and similarity between truncating and non-truncating *MYBPC3* pathogenic variant groups. Data is shown in violin plots with median and interquartile range. **B.** Average age-adjusted left atrial diameter was smaller among non-truncating pathogenic variant carriers. **C.** Broad variability in disease severity is reflected by range in age of diagnosis in both *MYBPC3* groups, with a modestly lower average age of diagnosis among non-truncating pathogenic variant carriers. **D.** Kaplan-Meier survival analysis shows no difference in the composite adverse event rate from time of birth between truncating and non-truncating pathogenic variant groups. Composite outcome consisted of first occurrence of any of the following: sudden cardiac death, resuscitated cardiac arrest, appropriate implantable cardioverter-defibrillator therapy, cardiac transplantation, LV assist device implantation, LV ejection fraction <35%, or New York Heart Association class III/IV symptoms, atrial fibrillation (AF), stroke, or death.

Circulation: Genomic and Precision Medicine

Figure 2. *MYBPC3* Truncating Pathogenic variants Cause Similar Phenotypic Severity Regardless of Variant Locus or Type. **A-B.** Truncating *MYBPC3* variants were categorized by locus quartiles within the gene to examine whether N-terminal or C-terminal truncations exert different effect sizes. No difference in extent of hypertrophy (A) or left atrial diameter (B) are observed. **C-D.** Four founder populations within SHaRe were compared to determine whether phenotypic severity is different in the setting of these 4 distinct truncating variant types

(c.742G>A = exonic splice variant, c.2373insG = frameshift, c.2827C>T = nonsense, c.2864_2865del = frameshift). No difference was observed either in the variance/distribution of hypertrophy (C) or in the magnitude of hypertrophy (D).

Figure 3. Distribution of *MYBPC3* pathogenic variants, variants of unknown significance, and common gnomAD variants relative to MyBP-C protein domains. Truncating *MYBPC3* pathogenic variants are dispersed homogeneously throughout the gene, while non-truncating pathogenic variants exhibit clustering in the C3, C6, and C10 domains (18 of 22, 82%). Non-truncating variants of unknown significance are dispersed throughout the gene, as are gnomAD common variants (i.e. allele frequency >4x10^{-5}).

Circulation: Genomic and Precision Medicine

Figure 4. Non-Truncating MyBP-C Mutant Protein Localizes to the Myofilament for C3 and C6 Domain Mutants but Does Not Incorporate into Myofilaments for C10 Domain Mutants. To determine whether mutant MyBP-C proteins integrate normally into the myofilaments, both FLAG-tagged control and mutant constructs were cloned into an adenoviral vector that was then used to transduce neonatal rat ventricular myocytes (NRVMs). 48 hours following transduction, NRVMs were immunofluorescently labeled with an anti-MyBP-C antibody to detect both endogenous and exogenously expressed MyBP-C (left column) and an anti-FLAG antibody to detect only the transduced MyBP-C (middle column). This system achieved stable integration of FLAG-control MyBP-C into myofilaments (top row) with no FLAG signal detected without viral transduction (second row). Non-truncating mutant MyBP-C for C3 and C6 domain pathogenic variants exhibited normal myofilament integration while C10 mutant MyBP-C exhibited poor or no myofilament localization.

Figure 5. Non-Truncating Mutant MyBP-C Degradation Rates Measured by Cyclohexamide Pulse Chase Demonstrate Rapid Degradation for C10 Domain Non-Truncating Mutant MyBP-C. To determine whether non-truncating *MYBPC3* pathogenic variants alter protein stability, NRVMS were transduced with adenoviral constructs expressing wild-type (WT) control and non-truncating mutant MyBP-C. Cyclohexamide was administered at 0, 30 minutes, 1 hour, 3 hours, 6 hours, and 12 hours to inhibit protein synthesis and MyBP-C was measured (see Methods). Data from two or more independent experiments performed in quadruplicate were fit to a first order exponential decay curve. The same control data (from FLAG-labeled wild type expressed MyBP-C) is depicted on each graph (A-C). **A-B.** C3 and C6 mutant MyBP-C demonstrates similar degradation rates as control. **C.** C10 mutant MyBP-C demonstrates rapid degradation compared to control. Data is represented as mean $\pm$ standard error of the mean. The calculated half-lives with 95% confidence intervals are shown in Table 2.

American Heart Association.
Circulation: Genomic and Precision Medicine

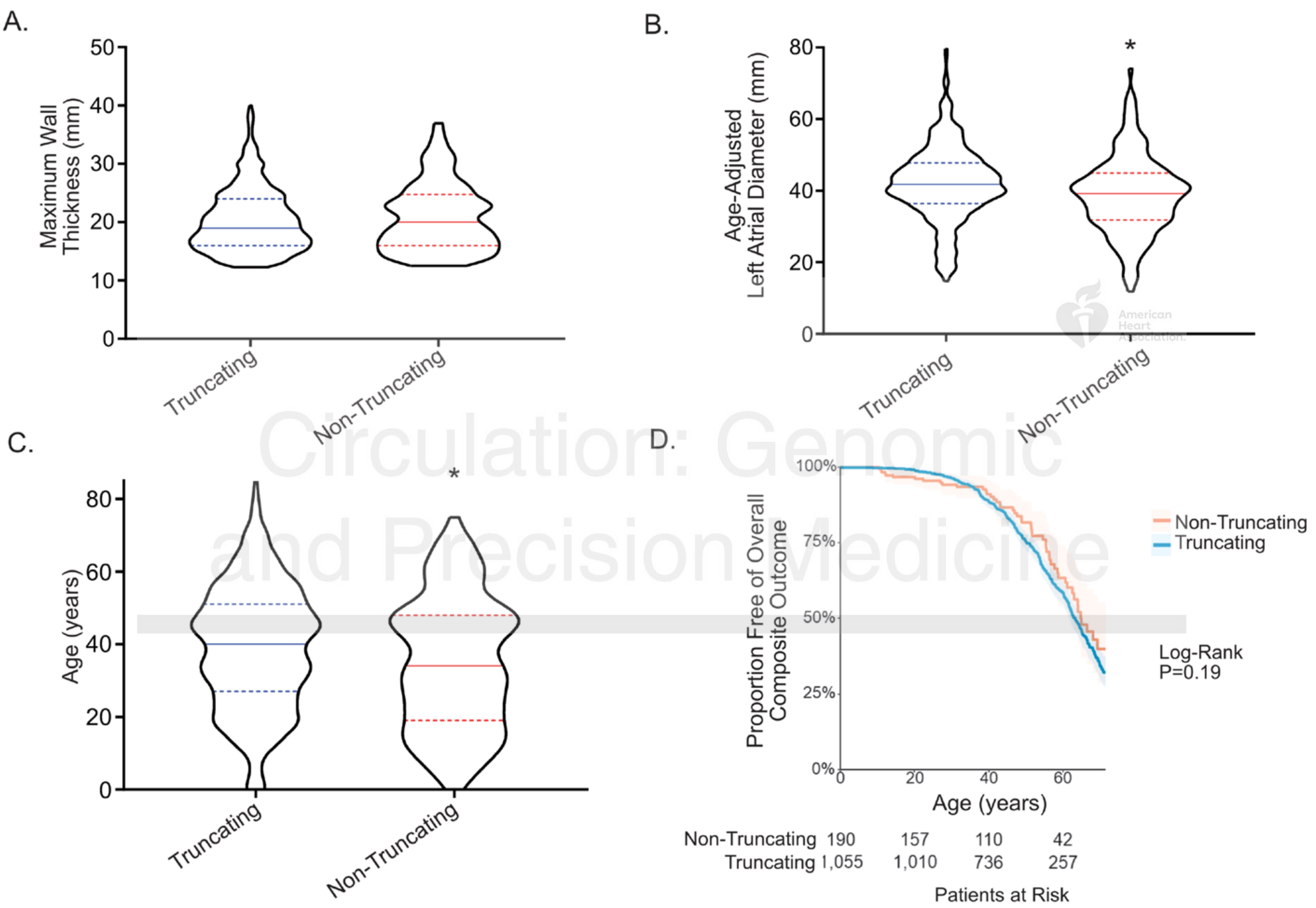

Circulation: Genomic and Precision Medicine

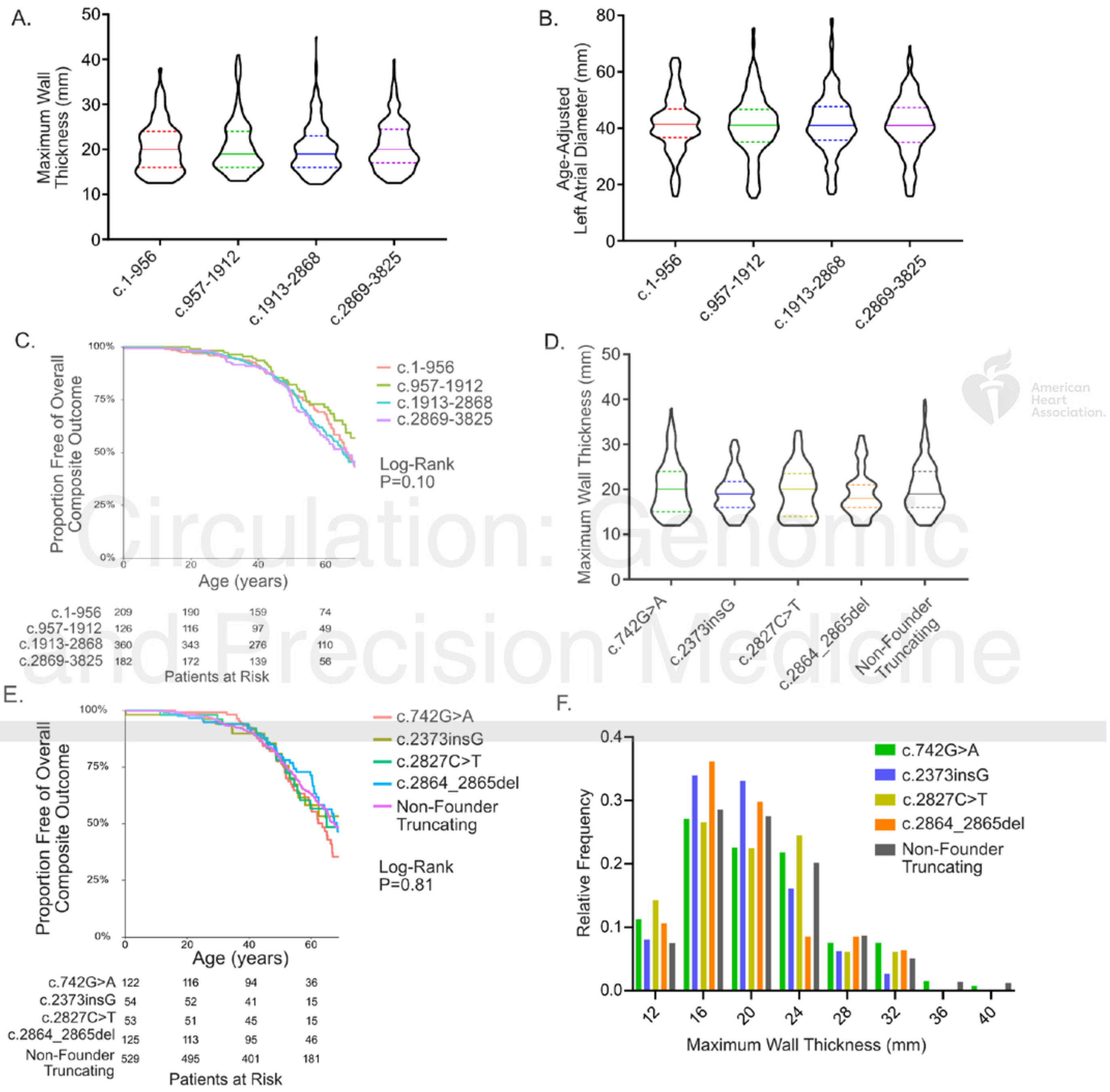

American Heart Association.
Circulation: Genomic and Precision Medicine

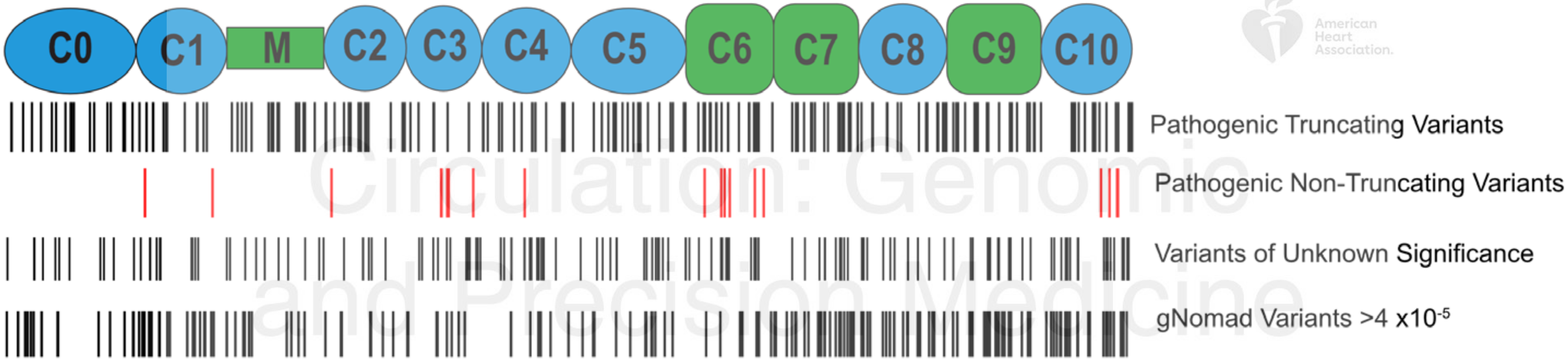

American Heart Association.
Circulation: Genomic and Precision Medicine

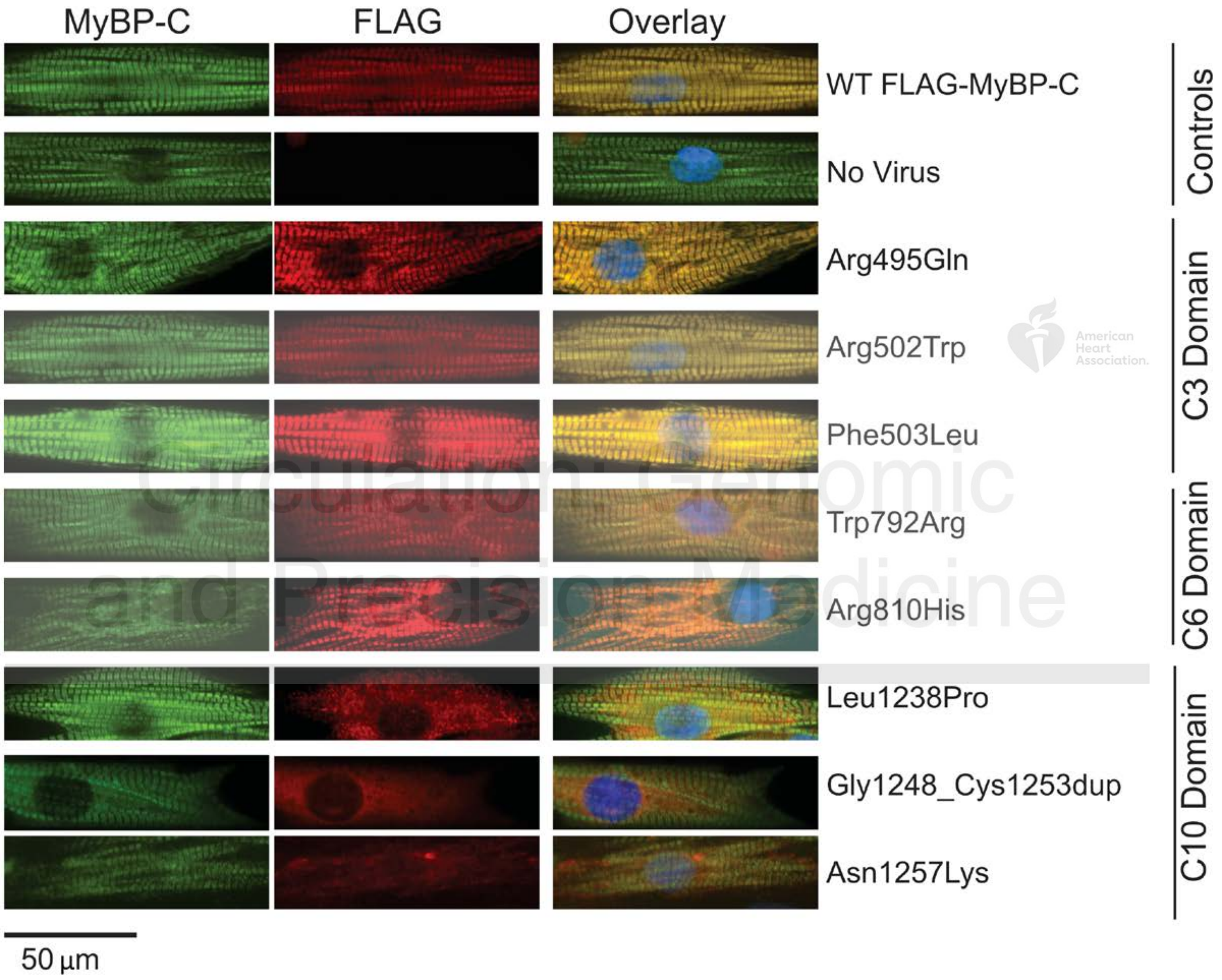

American Heart Association.
Circulation: Genomic and Precision Medicine

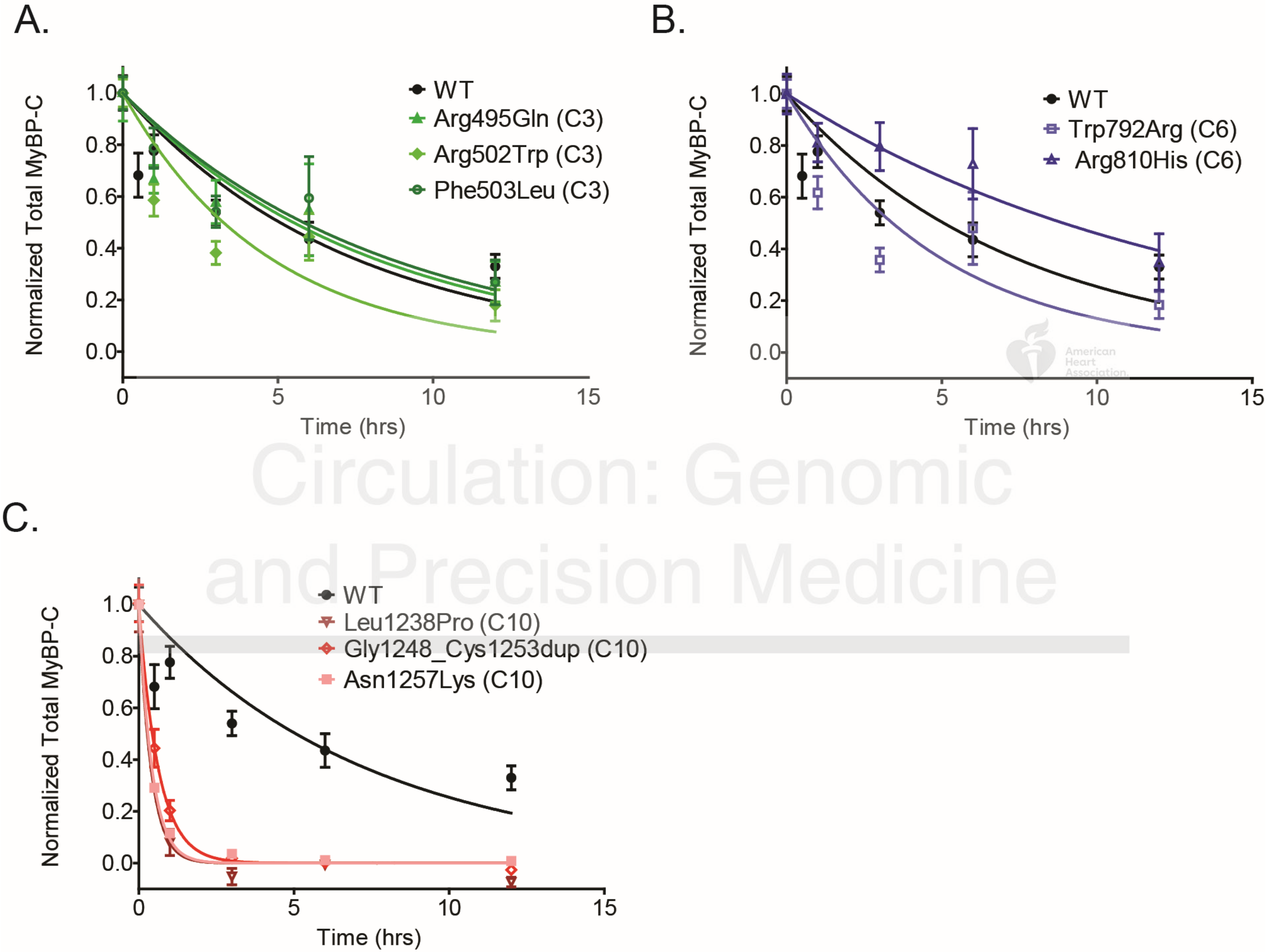

Circulation: Genomic and Precision Medicine
American Heart Association.
